# Methylenelactide: vinyl polymerization and spatial reactivity effects

**DOI:** 10.3762/bjoc.12.232

**Published:** 2016-11-14

**Authors:** Judita Britner, Helmut Ritter

**Affiliations:** 1 Institute of Organic Chemistry and Macromolecular Chemistry, Heinrich-Heine-University, Universitätsstraße 1, 40225 Düsseldorf, Germany

**Keywords:** copolymerization, kinetic study of the radical homopolymerization, push–pull monomer, reversible addition fragmentation chain transfer (RAFT)

## Abstract

The first detailed study on free-radical polymerization, copolymerization and controlled radical polymerization of the cyclic push–pull-type monomer methylenelactide in comparison to the non-cyclic monomer α-acetoxyacrylate is described. The experimental results revealed that methylenelactide undergoes a self-initiated polymerization. The copolymerization parameters of methylenelactide and styrene as well as methyl methacrylate were determined. To predict the copolymerization behavior with other classes of monomers, *Q* and *e* values were calculated. Further, reversible addition fragmentation chain transfer (RAFT)-controlled homopolymerization of methylenelactide and copolymerization with *N*,*N-*dimethylacrylamide was performed at 70 °C in 1,4-dioxane using AIBN as initiator and 2-(((ethylthio)carbonothioyl)thio)-2-methylpropanoic acid as a transfer agent.

## Introduction

Methylenelactide (MLA) with the IUPAC name (6*S*)-3-methylene-6-methyl-1,4-dioxane-2,5-dione is a radically polymerizable vinyl-lactide derivative. The molecule’s quaternary carbon atom located at the double bond is substituted with an electron withdrawing (“pulling”) carbonyl group and an electron donating (“pushing”) oxygen atom. Monomers with such substitution patterns are defined as captodative or push–pull monomers [1]. MLA was first synthesized in 1969 by Scheibelhoffer et al. through a bromination of L-lactide followed by a basic HBr elimination [2]. In 2008, the first Diels–Alder reaction employing MLA as dienophile was described [3–6]. In a recent NMR study we demonstrated that, poly(MLA) prepared via free radical polymerization contains mainly isotactic units. Furthermore, we found that the polymer attached lactide rings react like activated esters and thus readily undergo quantitative amidation reactions with aliphatic primary amines under mild conditions [7]. In the underlying study, we focused on spatial effects with respect to interactions between neighboring lactide rings. Based on these findings, polymer analogous reactions of poly(MLA) with different alcohols were recently investigated [8]. Up to now, it was not possible to polymerize MLA via ring opening [9]. Only indirectly, unsaturated polylactide carrying vinyl side groups can be obtained through a copolymerization of chlorolactide with L-lactide followed by subsequent dehydrochlorination [10]. Recently, thiol-Michael additions on MLA were reported [11–12].

In this paper, we wish to present a kinetic study of free radical and controlled/living radical polymerization of MLA. The latter reactions were conducted via a reversible addition fragmentation chain transfer (RAFT) mechanism. We also investigated the copolymerization of MLA with styrene and methyl methacrylate, respectively. The results were compared to the well-known push–pull type monomer α-acetoxyacrylate.

## Results and Discussion

### Free-radical polymerization of methylenelactide MLA

**Figure 1:**
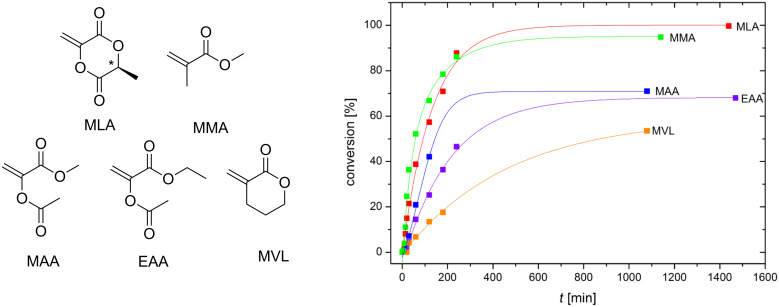
Structures of used monomers and the time-conversion plot of the corresponding free-radical polymerization reactions (80 wt % DMF, 1 mol % AIBN, 70 °C).

The push–pull type monomer MLA contains an electron-deficient vinyl group which is structurally related to acrylate monomers. Electron-rich vinyl groups are structurally related to vinyl ester monomers. However, the free-radical polymerization of MLA proceeds smoothly at elevated temperature without ring-opening side reactions (see Figure S1 in Supporting Information File 1). To evaluate the free-radical polymerization of MLA, we compared the behavior to non-cyclic, pull-type methyl methacrylate (MMA), non-cyclic, push–pull-type methyl α-acetoxyacrylate (MAA) and ethyl α-acetoxyacrylate (EAA), respectively and cyclic pull-type α-methylene-δ-valerolactone (MVL, see Figure 1).

Since the polymerization kinetics are mainly controlled by steric effects and the polarity of the double bonds, we evaluated the electronic structure of the different monomers via ^1^H nuclear magnetic resonance (NMR) spectroscopy. As expected, the double bond protons of MLA at 5.77 and 5.56 ppm clearly differ from the double bond protons of MAA (6.02 and 5.65 ppm) and EAA (5.99 and 5.62 ppm). Surprisingly, their chemical shifts are very similar to the double bond protons of MMA (6.03 and 5.66 ppm). This suggests that the electron-withdrawing substituent has a stronger influence on the electron density of the vinyl protons than the electron-pushing substituent (Table S1, Figures S2 and S3, Supporting Information File 1). We further employed ^13^C NMR spectroscopy to provide a better view on the electron density of the double bond. It turned out that the quaternary carbon atoms of the double bond of EAA (144.31 ppm), MAA (144.04 ppm) and MLA (143.69 ppm) experience a stronger impact through the electron-withdrawing substituent than the corresponding carbon atoms of MMA (135.77 ppm) and MVL (134.09 ppm). The electron-pushing substituent influences preferentially the methylene carbon atom. This methylene carbon atom shows a relatively high electron density in case of MLA (108.31 ppm), MAA (114.67 ppm,) and EAA (114.32 ppm) compared to the lower electron density in MMA (125.59 ppm) and MVL (127.74 ppm) (Table S1 and Figure S3, Supporting Information File 1).

The homopolymerization reactions were carried out in presence of 1 mol % of AIBN at 70 °C. The conversion after different reaction times was determined via ^1^H NMR spectroscopy (Figure 1). The molecular weights and dispersities (*Đ*) of the obtained polymers are summarized in Table S2 (Supporting Information File 1).

Interestingly, the polymerization kinetics of MLA are similar to these of MMA. In contrast, the non-cyclic push–pull type monomers MAA and EAA are both less reactive. This indicates that in addition to steric hindrance, the mobility of the substituents plays an important role in the spatially controlled chain growth reactions. The molecular weights (*M*_n_) are 21 600 g mol^−1^ for poly(MAA) and 31 600 g mol^−1^ for poly(EAA) with narrow dispersities (*Đ*) between 1.5 and 1.7, indicating that chain termination mainly occurs through recombination of polymer radicals [13].

The moderate conversion of MVL is presumably a result of the relatively low ceiling-temperature of the corresponding polymer (at 81 °C) [14]. This means that under the applied reaction conditions the rate of the polymerization reaction is only slightly higher than the depolymerization rate, which results in slow polymer growth. The obtained data also indicates that the electron densities of the vinyl groups of the used monomers play a minor role with respect to the polymerization kinetics. The higher mobility of the free substituents of the non-cyclic push–pull type monomers MAA and EAA causes a reduced polymerization rate (Figure 1) compared to that of the stiff cyclic molecule MLA.

### Stereochemistry of poly(MLA)

**Scheme 1:**
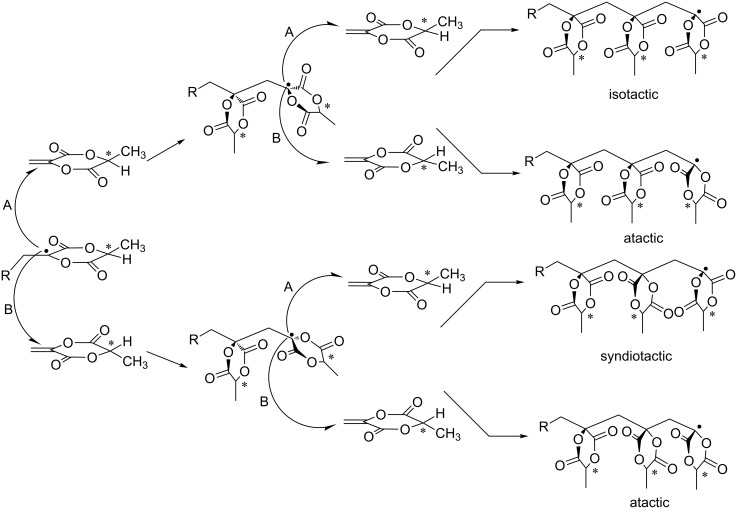
Stereospecific propagation of chiral MLA illustrating the triade formation [15].

As we reported recently, MLA polymerizes via free-radical polymerization to yield predominantly isotactic polymer structures (Figure S4, Supporting Information File 1). Similar findings were reported by Tanaka et al. who investigated the polymerization of methylene dioxolanone derivatives yielding predominantly isotactic polymers [15]. Our recently reported spatial dipole–dipole interactions between neighboring lactide units were supported by IR spectroscopy, as the interactions causes two separate carbonyl stretching vibrations. This effect may also play a crucial role in the isotactic propagation steps during MLA polymerization [7]. In contrast, the polymer of non-cyclic MAA shows a preferred syndiotactic (*rr*) conformation caused by steric control of the free substituents as indicated by ^13^C NMR spectroscopy (Figure S5, Supporting Information File 1). Scheme 1 shows the different potential propagation steps of MLA.

### Deviation of classical polymerization kinetics of MLA

**Figure 2:**
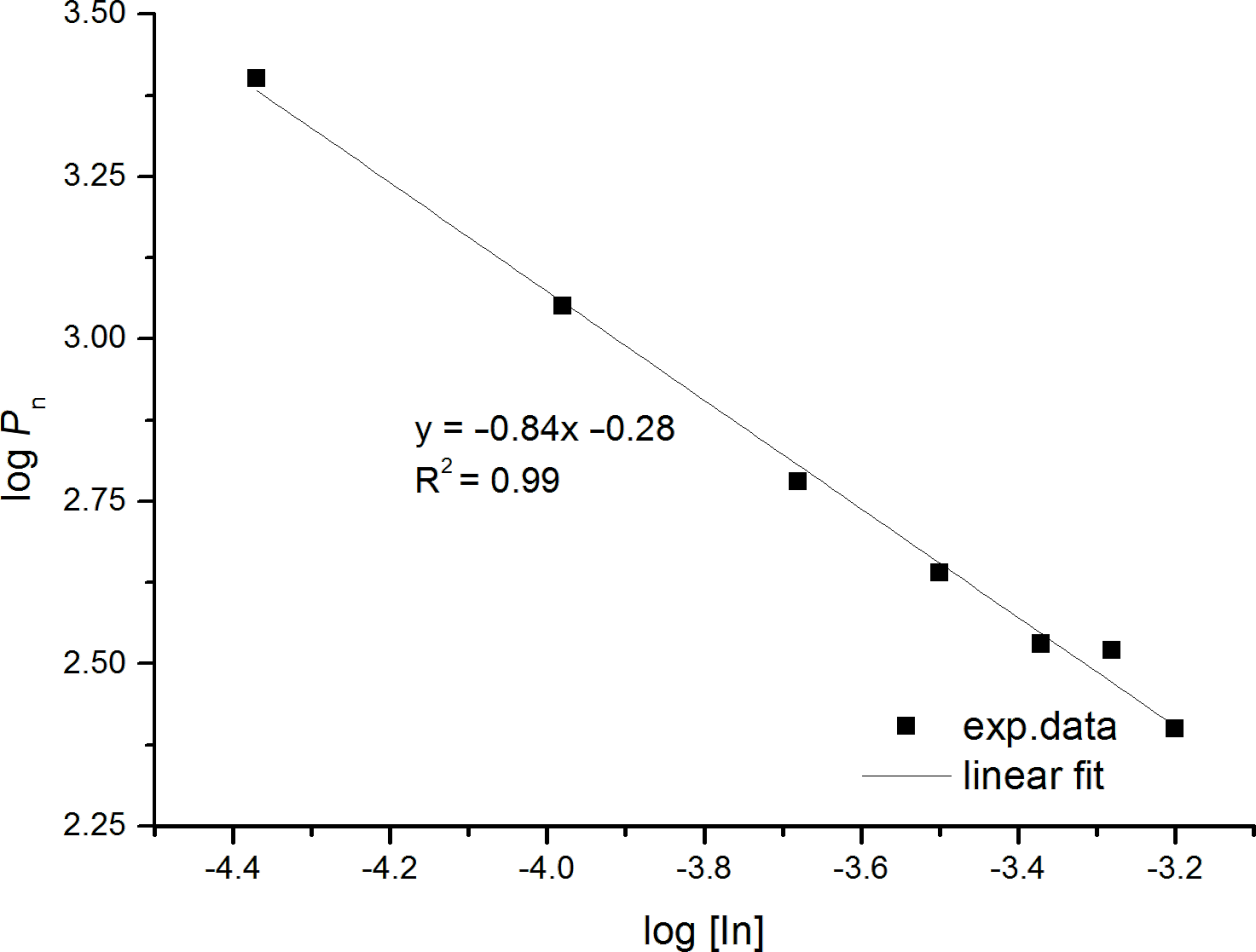
Plot of log *P*_n_ versus log [In] of the polymerization of MLA with different mol % AIBN.

Usually, the rate of polymerization is proportional to the square root of initiator concentration [In] and the degree of polymerization (*P*_n_) is inversely proportional to the square root of [In]. To investigate the polymerization behavior of MLA at 70 °C, different molar amounts of AIBN were used. The polymerization reactions were evaluated after ca. 2 minutes at low conversions up to 10% as determined by ^1^H NMR spectroscopy. The precipitated polymers were analyzed by size exclusion chromatography (SEC) in DMF (Table 1). The logarithmic plot displayed in Figure 2 shows the correlation between the degree of polymerization and the initiator concentration. The slope was determined to be −0.84, which significantly deviated from the expected value of 0.5. This observation indicates some self-initiation beside AIBN initiation.

**Table 1:** SEC data from the polymerization of MLA with different amounts of AIBN (*c*_(MLA)_ = 1.812 mol L^−1^in 1,4-dioxane, 15–1 mol % AIBN, 70 °C, polymerization time 2 minutes).

sample	1	2	3	4	5	6	7

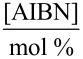	15	12.5	10	7.5	5	2.5	1
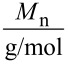	35 600	46 800	47 600	62 200	85 400	158 800	358 200
*Đ*	1.8	5.7	4.6	3.4	5	2.7	2.4

### Self-initiation of MLA

**Scheme 2:**
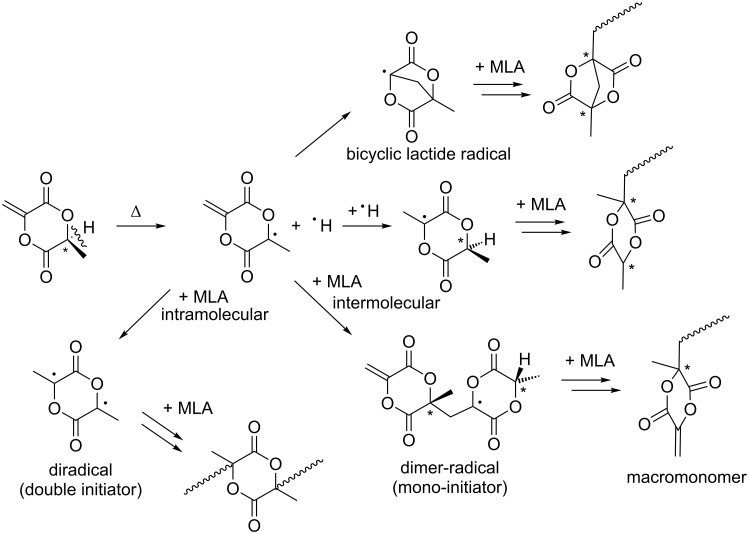
Postulated mechanism of the self-initiation of MLA.

**Figure 3:**
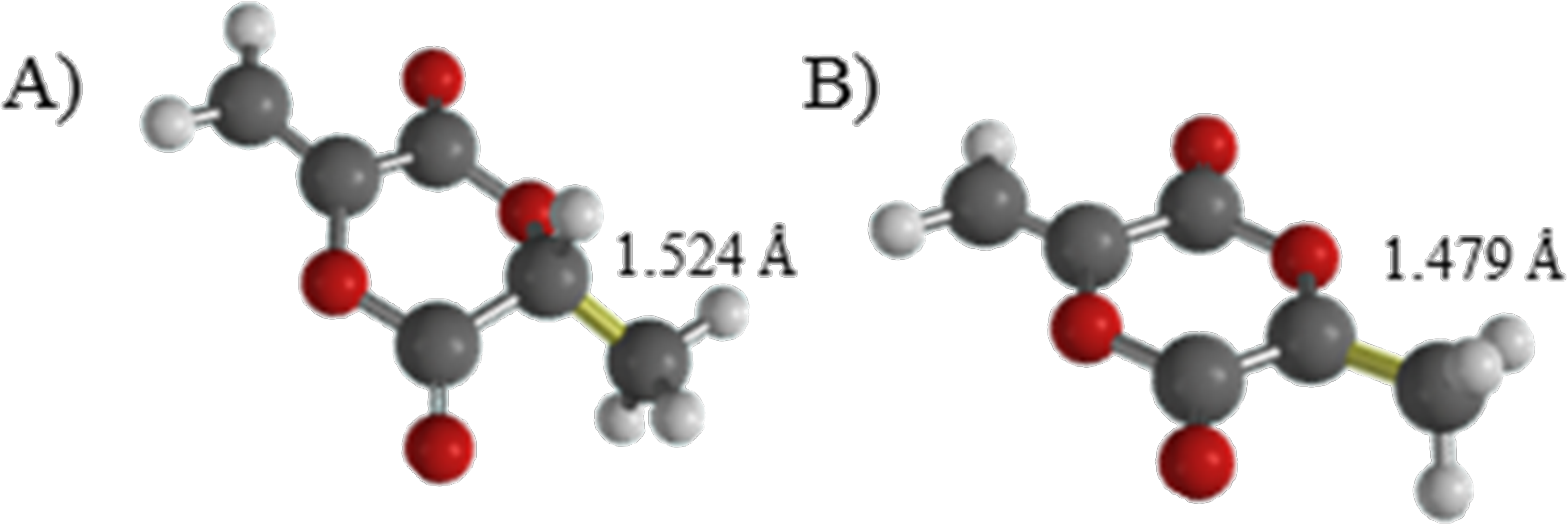
DFT-calculated C–C binding length (yellow) of (A) MLA and (B) the corresponding radical.

The self-initiation of some non-cyclic push–pull monomers is already known [1]. However, up to now, the free radical self-initiation of cyclic MLA has not been described in the literature. Thus, we herewith show our postulated mechanism for the self-initiation of MLA in Scheme 2. We propose that a homolytically H–C cleavage takes place in a first step yielding two radicals. This process is accompanied by a change of hybridization from a tetrahedral sp^3^ structure of the chiral center to a trigonal planar sp^2^ structure of the resulting radical. Scheme 2 also shows additional postulated radical reactions including the formation of a bicyclic lactide radical to initiate the main polymerization. Since the spontaneous homolytically C–H cleavage may represent the first step in the reaction cascade, theoretical calculations on a DFT level were conducted. The above mentioned hybridization change as driving force for C–H cleavage is verified in the reduced bond length of the C–CH_3_ bond from 1.542 Å (MLA) to 1.479 Å for the corresponding radical. This clearly indicates a stabilization of this C–C bond after C–H cleavage (Figure 3).

Since only soluble polymers were obtained, the C–H bonds in the linear MLA-polymer units must be more stable than in the monomeric MLA. Otherwise, crosslinking should take place via spontaneous C–H cleavage and chain recombination. This important point could be verified by IR spectroscopy and also by theoretical calculations of the force constants of the C–H bonds on a DFT level.

**Figure 4:**
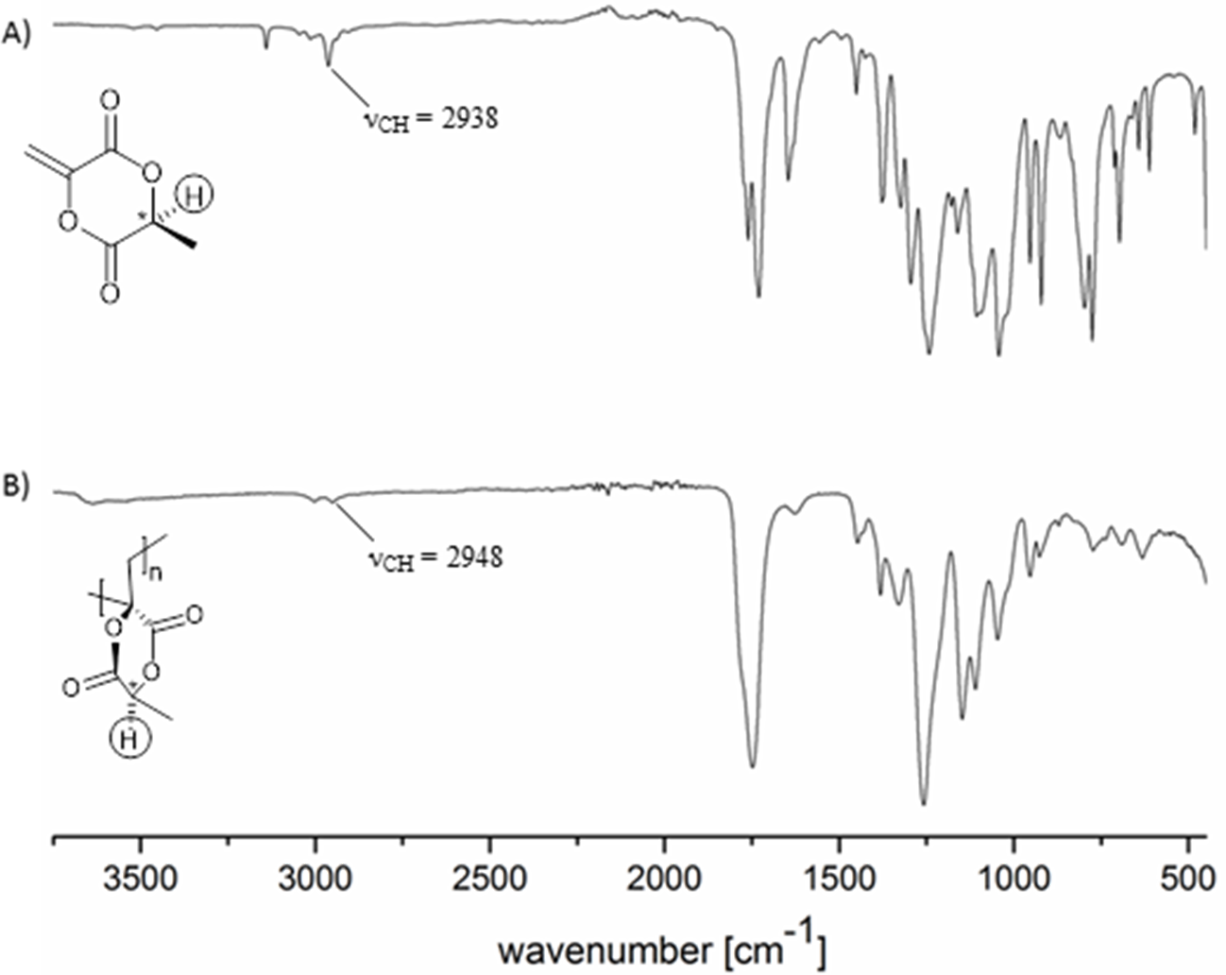
IR spectra of (A) MLA and of (B) poly(MLA) prepared by the self-initiated polymerization at 70 °C.

The C–H stretching vibrations ν_(C-H)_ = 2948 cm^−1^ of poly(MLA) determined via IR spectroscopy correlate well with the force constant of *k* = 473 N m^−1^ (calculations see Figure S6, Supporting Information File 1). In contrast, the monomer MLA (ν_(C-H)_ = 2938 cm^−1^) has a significantly lower force constant of *k* = 467 N m^−1^. This also gives a strong hint on the postulated relatively easy C–H homolytical cleavage from MLA as described in Scheme 2. This measured IR values correspond nicely to the DFT calculations (poly(MLA) ν_(C-H)_ = 2922 cm^−1^, MLA ν_(C-H)_ = 2914 cm^−1^). Figure 4 shows the IR spectra of MLA and of the obtained poly(MLA).

**Figure 5:**
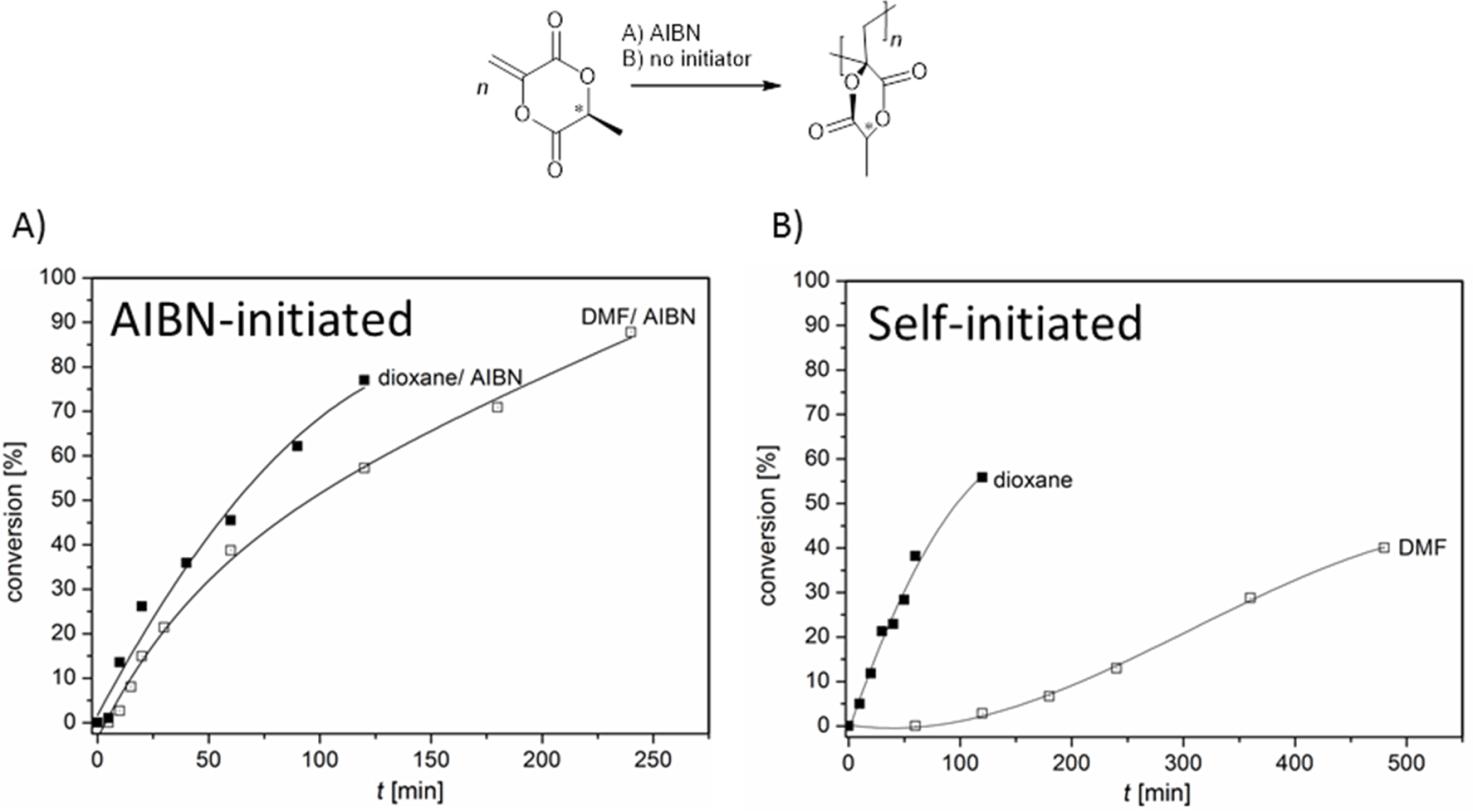
Conversion plot of the polymerization of MLA in 1,4-dioxane and DMF (*c*_MLA_ = 1.8 mol L^−1^, *c*_AIBN_ = 1.8 × 10^−2^ mol L^−1^, 70 °C) with AIBN (A) and without initiator (B).

To evaluate some kinetic solvent effects of the discussed self-initiated polymerization reactions of MLA, the kinetics of the AIBN-initiated and initiator-free radical polymerizations of MLA were repeated in less polar 1,4**-**dioxane and dipolar DMF as solvents (Figure 5). Surprisingly, the yields of self-initiated polymerization in 1,4-dioxane are very similar to the yields of AIBN-initiated polymerization. In contrast, the self-initiation polymerization of MLA is much more retarded in DMF solution than in 1,4-dioxane. Taking our postulated radical formation process into account, the dipolar solvent DMF stabilizes the polar educt MLA more than the less polar 1,4-dioxane. Since the formed radical is planar and less polar, the activation energy to this radical formation must be higher in DMF than in 1,4-dioxane [16]. Interestingly, the self-initiated poly(MLA) has a relatively high molecular weight of *M*_n_ = 180 000 g mol^−1^ (*Đ* = 2.5) compared to the AIBN initiated poly(MLA) (*M*_n_ = 73 000 g mol^−1^, *Đ* = 2.6). A self-initiated poly(MLA) obtained at 30 °C yields with a reduced molar mass of *M*_n_ = 28 600 g mol^−1^, *Đ* = 1.9 (Figure S7, Supporting Information File 1). Poly(MLA) polymerized in DMF could not be analyzed by SEC because of some unknown side products (Figure S8, Supporting Information File 1).

For comparison, the non-cyclic MAA shows even in bulk only a very low yield of ca. 10 mol % of self-initiated polymer at 60 °C [13,17]. Thus, the ring shaped MLA is much more reactive in respect to the self-initiated polymerization.

### Calculated initial rate for the self-initiated polymerization of MLA by the use of DPPH

**Figure 6:**
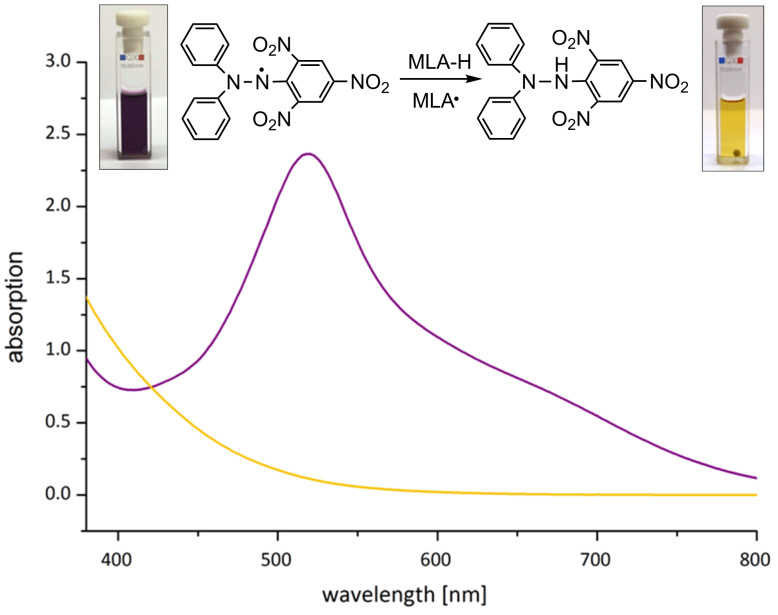
UV–vis spectra of the reaction mixture with DPPH radical at the beginning (violet line, 0.23 mM) of the self-initiated polymerization of MLA and after 15 h (yellow line) in a range from 380–800 nm (*c*_MLA_ = 1.8 mol L^−1^, 70 °C).

As discussed above, the formation of free radicals is a key step for spontaneous polymerization of MLA. Accordingly, spontaneously formed radicals can be proved by the use of the strongly colored 1,1-diphenyl-2-picrylhydrazyl radical (DPPH) which reacts with H radicals under decolorization. The consumption of DPPH-radicals can be followed by the naked eye. Figure 6 shows the UV–vis absorption spectra of DPPH from the beginning of the self-initiated polymerization at 70 °C and after 15 h.

The concentration of DPPH plotted against the time at 70 °C and 30 °C gives a straight line indicating that the reaction follows pseudo zero-order kinetics (Figures S10 and S11, Supporting Information File 1). The slope of this plot corresponds to the reaction rate. The reaction rate of disappearance of DPPH (*R*_DPPH_) is equal to the value of the rate of MLA self-initiation (*R*_i_). Accordingly, at 70 °C the self-initiated polymerization with a rate of 2.4 × 10^−4^ mM s^−1^ is 5 times higher than at 30 °C with a rate of 4.42 × 10^−5^ mM s^−1^ (Figure S12, Supporting Information File 1). The actual polymerization reaction takes place after DPPH was consumed, since the molecule acts as an inhibitor. In a control experiment performed in absence of MLA, the DPPH concentrations remained stable.

### Free radical copolymerization behavior of MLA

The copolymerization parameters of MLA with styrene and MMA, respectively were evaluated through the method of Kelen and Tüdös [18]. For this, the residual monomer ratio was determined by high performance liquid chromatography (see execution, characterization methods and Figures S14 and S15 in Supporting Information File 1).

The copolymerization parameters obtained from the MLA and styrene system were *r*_1_ = 0.8 (MLA) and *r*_2_ = 0.7 (styrene) which indicates that the copolymerization process proceeds partially alternating. The Alfrey–Price *Q* and *e* values were also calculated from the experimental data. The values for MLA are *Q* = 0.79 and *e* = 0.015 (see Figure S16 for *Q* and *e* value calculation, Supporting Information File 1) [19]. The constant *Q* reflects the resonance stabilization of the growing radical. Large *Q* values (>0.5) indicate stabilized monomers. The constant *e* reflects the polarity of the double bond and of the growing radical. For instance positive *e* values point to an electrophilic character while negative *e* values point to a nucleophilic character.

In contrast, the non-cyclic monomers MAA and EAA show higher positive *e* values and are thus highly influenced by the pull substituents. These higher *e* values are also indicated in the ^13^C NMR data described above and by higher dipole moments in MAA (3.79 Debye) and EAA (2.26 Debye) compared to MMA (4.10 Debye) and MLA (2.09 Debye) (refer to Table S1, Supporting Information File 1).The *Q* and *e* values of various monomers are summarized in Table 2 [20–22].

**Table 2:** Alfrey–Price *Q* and *e* values of various monomers with styrene as reference system.

Monomer	*Q*	*e*

styrene	1	−0.8
MLA	0.79	0.015
MMA	0.78	0.40
MAA	1.65	0.57
EAA	0.52	0.77
vinyl acetate	0.026	−0.88
*N,N*-dimethylacrylamide (DMAa)	0.55	−0.56

**Figure 7:**
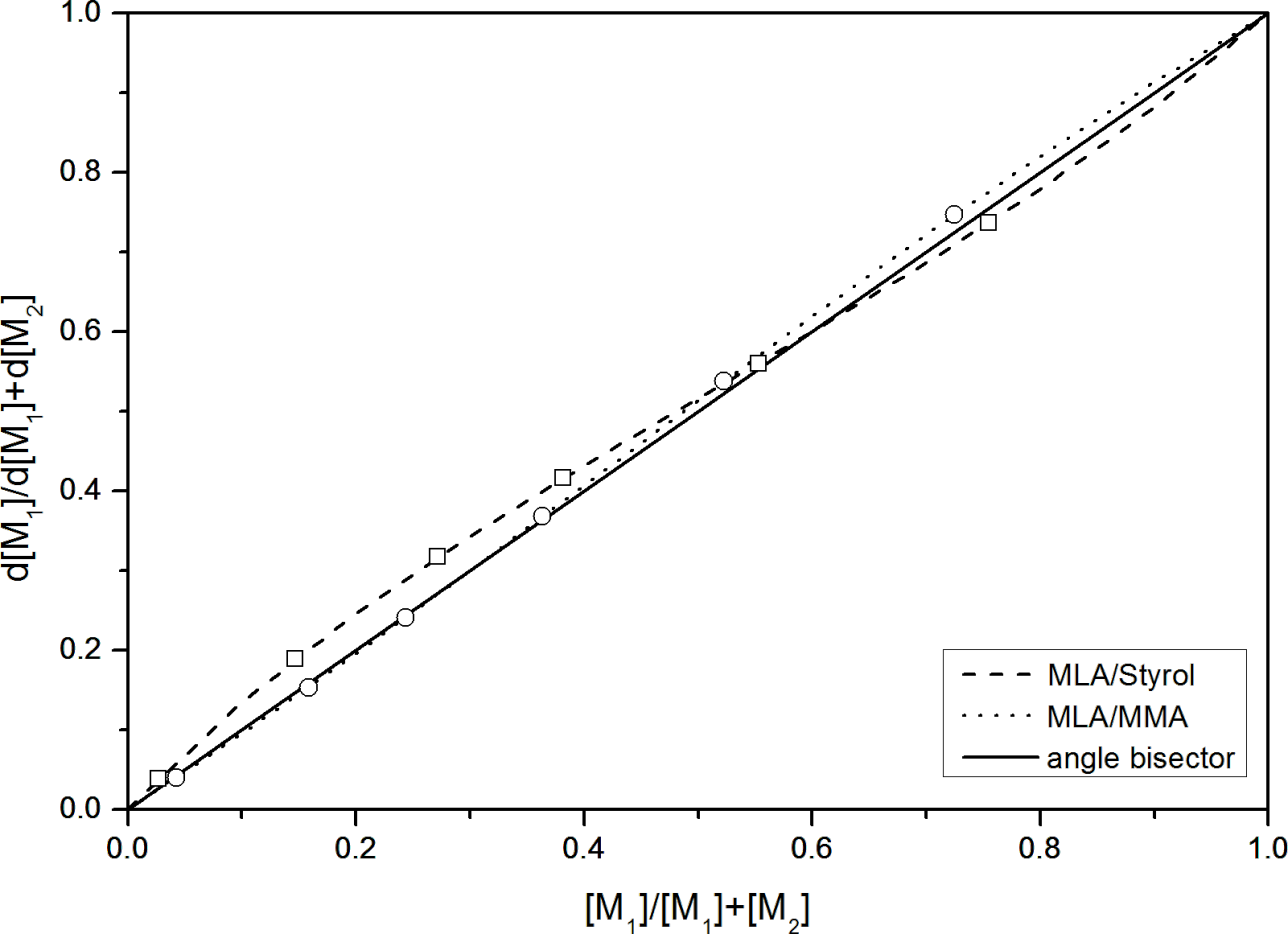
Copolymer composition curves for the systems MLA with styrene and MMA.

The copolymerization parameters obtained from MLA and MMA were *r*_1_ = 1.1 (MLA) and *r*_2_ = 1.2 (MMA) which indicate an almost statistical process, with a slight tendency to homoadditon. Figure 7 illustrates the obtained copolymer composition curves for the systems of MLA with styrene and MMA, respectively.

### Chain-transfer agents for free-radical polymerization

Attempts to reduce the molecular weight during the MLA polymerization by the use of classical chain-transfer agents such as mercaptoethanol, mostly failed (Figure S17 and Table S7, Supporting Information File 1). A preferred nucleophilic attack of the thiol takes place**.** This can be clearly seen in the ^1^H NMR spectra (Figures S18 and S19, Supporting Information File 1).

**Figure 8:**
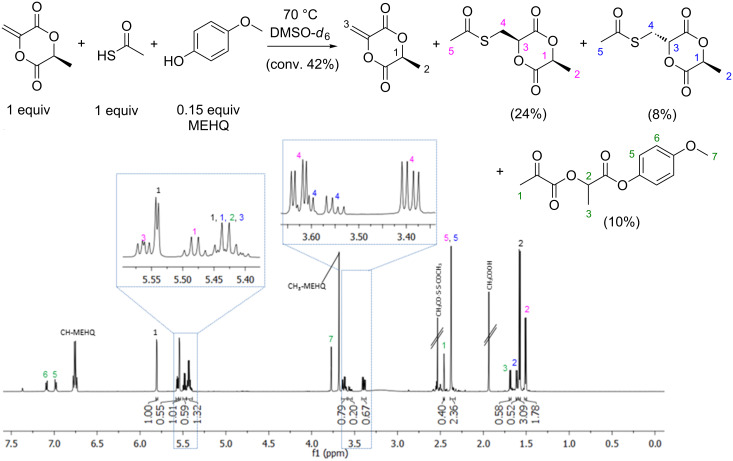
^1^H NMR spectrum of MLA with 1 equiv of thioacetic acid and 0.15 equivalents of an inhibitor 4-methoxyphenol (MEHQ) measured after 30 min at 70 °C in a NMR spectrometer (600 MHz, DMSO-*d*_6_, 70 °C, *c*_MLA_ = *c*_Thioacetic acid_ 0.5 mol∙L^−1^).

Thioacetic acid was used as a potential chain-transfer agent due to its lower nucleophilicity. However, a complete thiol-Michael addition can be seen in Figure 8 (not full conversion of MLA due to the impurities of thioacetic acid like disulfide and acetic acid). In this context, the iodine catalyzed thiol-Michael addition was investigated [11].

### Controlled radical polymerization of MLA via RAFT

**Scheme 3:**
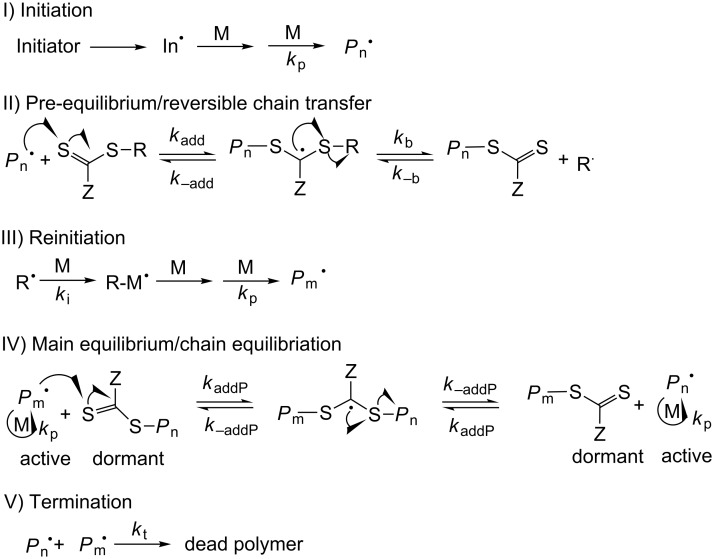
Mechanism of RAFT polymerization [24].

Since MLA acts as a vinyl monomer, it was also interesting to evaluate the controlled RAFT mechanism. Recently, the MADIX (macromolecular design via the interchange of xanthates) technique was found to be unsuccessful for the controlled radical homopolymerization of the non-cyclic monomer EAA. Only in the presence of acrylic monomers copolymerization of EAA under MADIX conditions was possible [23]. For MLA polymerization under controlled radical conditions, we evaluated a similar type of polymerization, the RAFT mechanism as shown in Scheme 3. The reversible series of addition and fragmentation between dormant and active chain ensure uniform growth of all chains with narrow dispersity (*Đ*).

**Figure 9:**
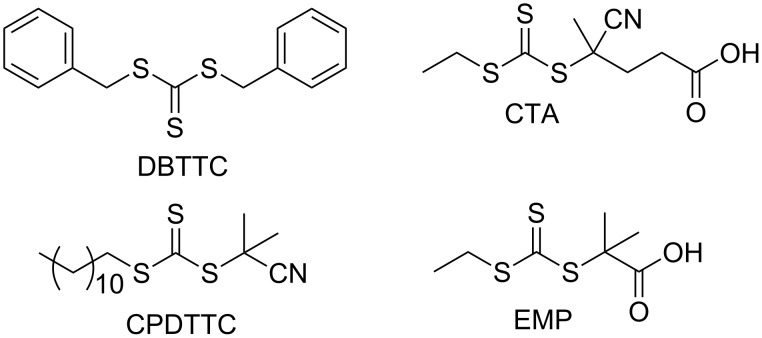
Structures of used RAFT agents examined in the polymerization of MLA.

The general structures of the RAFT agents contain a thiocarbonylthio group with reactive C–S double bond and attached R- and Z-group, whereas MADIX only refers to xanthates. Four RAFT agents with different polarities based on trithiocarbonate were examined in the RAFT homopolymerization of MLA (Figure 9).

The data of the RAFT homopolymerization of MLA are summarized in Table 3. Only in the presence of the more polar 4-cyano-4-(((ethylthio)carbonothioyl)thio)pentanoic acid (CTA) and 2-(((ethylthio)carbonothioyl)thio)-2-methylpropanoic acid (EMP) a polymerization took place. However only with EMP narrow dispersity was achieved (*Đ* = 1.6). This dispersity of 1.6 illustrates the upper limit for a successful RAFT process. Beside the good dispersity, the *M*_n_ in comparison to the theoretical value *M*_n theo._ is much higher due to the known parallel running process of self-initiation. For this reason, the polymerization with EMP was further examined.

**Table 3:** RAFT polymerization of MLA with different RAFT agents in a ratio of 98.87:1:0.125 ([MLA]/[RAFT]/[AIBN]) (80 wt % 1,4-dioxane, at 70 °C).

run	[1]/RAFT/[AIBN] [mol %]	Time [h]	Conversion [%]	*M*_n theo_^a^ [g/mol]	*M*_n SEC_^b^ [g/mol]	*Đ*

1	98.87/**DBTTC**/0.125	16	2.7	–	–	–
2	98.87/**CPDTTC**/0.125	16	4.2	–	–	–
3	98.87/**CTA**/0.125	18	87	12 500	55 400	2.3
4	98.87/**EMP**/0.125	16	>99	14 30	43 000	1.6

^a^Calculated theoretical molecular weights see characterization method in Supporting Information File 1. ^b^Determined by PS-calibrated SEC.

Upon further experiments with EMP (Table 4), the self-initiation becomes evident. Run 6 show the extent of the self-initiation with *M*_n_ of 17 200 g mol^−1^ and *Đ* = 2. A reduced amount of MLA result in bimolecular *M*_n_ (run 7, Table 4) and a doubling of the amount of MLA in much higher *M*_n_ (runs 8 and 9, Table 4), by the dominant part of self-initiation. The runs in dry DMF seem to be better in terms of *M*_n_, but in terms of dispersity, too high for the RAFT process (runs 10 and 11, Table 4). The isotacticity of the MLA polymers obtained in the RAFT polymerization was identical to those measured in the free-radical polymerization (Figure S20, Supporting Information File 1). The RAFT copolymerization with *N*,*N*-dimethylacrylamide (DMA) was investigated to reduce the self-initiated part [23].

**Table 4:** RAFT homopolymerization of MLA with EMP (80 wt % 1,4-dioxane at 70 °C).

run	[MLA]/[EMP]/[AIBN] [mol %]	Time [h]	Conversion [%]	*M*_n theo_^a^ [g/mol]	*M*_n SEC_^b^ [g/mol]	*Đ*

4	98.87/1/0.125	16	>99	14 300	43 000	1.6
5	98.87/0/0.125	17	>99	79 100	35 800	2.3
6	98.87/0/0	16	100	–	17 200	2.0
7	49.44/1/0.125	16	97	7 000	16 600	1.5
8	197.74/1/0.125	18	>99	28 300	191 300	2.4
9^c^	197.74/1/0.125	18	58	16 400	80 900	1.9
10^d^	197.74/1/0.125	20	92	26 200	18 500	1.9
11^d^	98.87/1/0.125	18	70	10 000	50 000	2.2

^a^Calculated theoretical molecular weights (see characterization methods in Supporting Information File 1). ^b^Determined by PS-calibrated SEC. ^c^180 wt % of 1,4-dioxane. ^d^80 wt % dry DMF as solvent.

### RAFT-Copolymerization of MLA with *N*,*N*-dimethylacrylamide (DMA)

Copolymerization of MLA with DMA was conducted aiming for copolymers with a molecular weight of *M*_n_ of 20 000 g mol^−1^. The results of the RAFT copolymerization are summarized in Table 5 (see SEC traces Figure S21, Supporting Information File 1).

**Table 5:** RAFT copolymerization of MLA with DMA (0.5 mol % EMP and 0.0625 mol % AIBN, 80 wt % 1,4-dioxane, 70 °C).

run	[DMA]/[MLA] [mol %] ^a)^	Time [h]	Conversion [%]^a)^	*M*_n theo_^a^ [g/mol]	*M*_n SEC_^b^ [g/mol]	*Đ*	*T*_g_ [°C]

12	50/50	18	43/91	17 400	31 200	1.6	193
13	75/25	18	100/100	22 200	29 300	1.3	149
14	85/15	18	72/100	16 600	22 300	1.3	139
15	90/10	18	100/100	20 900	28 800	1.2	131
16	95/5	18	63/100	13 400	22 200	1.3	127.
17	100/0	18	100	19 900	20 400	1.2	121

^a^Calculated theoretical molecular weights (see characterization methods in Supporting Information File 1). ^b^Determined by PS-calibrated SEC.

As expected, the *M*_n_ values come closer to the theoretical values, the more DMA is used (Figure S21, Supporting Information File 1). At runs 12, 14 and 16 (Table 5) the MLA revenues were not quantitative with a slightly lower dispersity may be due to a longer induction period, but this also occurred in the repetition in other runs.

**Figure 10:**
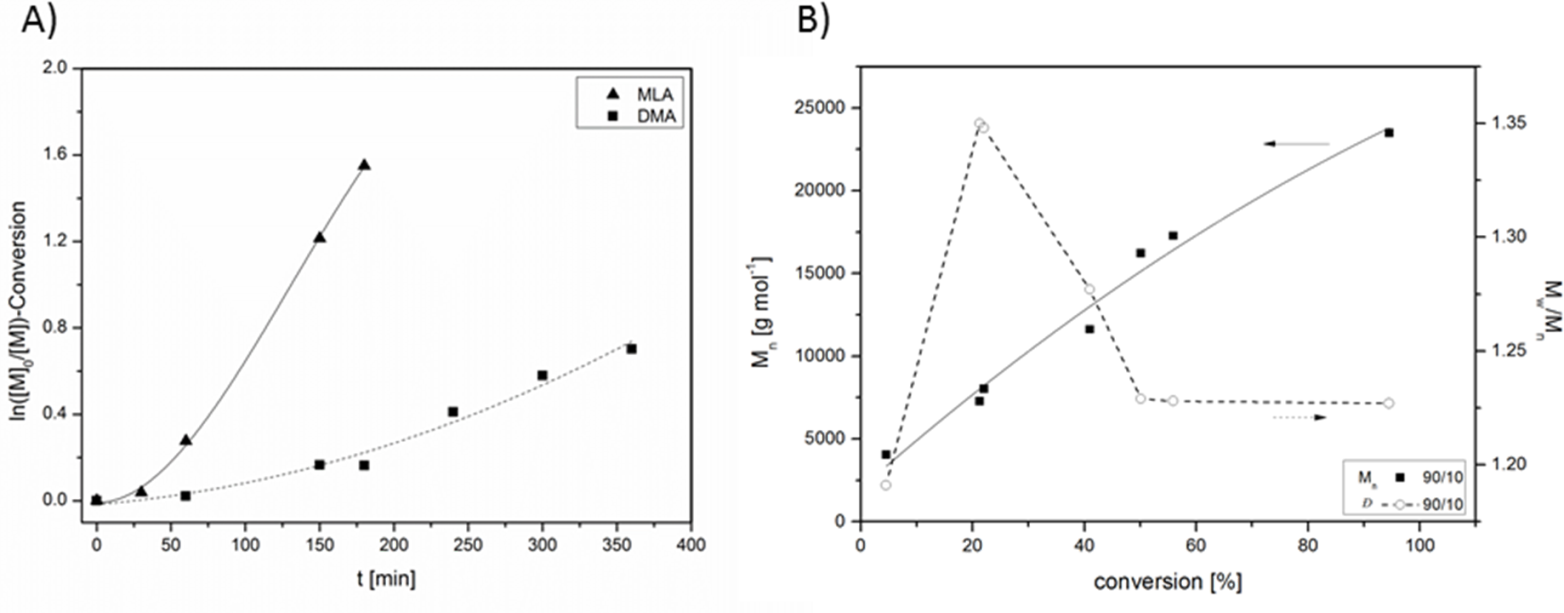
A) Kinetic plot for the RAFT copolymerization of MLA and DMA for the ratio 90/10 employing EMP. B) The evolution of *M*_n_ (full symbols) and *Đ* (empty symbols) with conversion of the copolymerization.

To investigate the process of the RAFT copolymerization of DMA with MLA the semi-logarithmic plot of conversion against time of run 15 (ratio 90/10, Table 5) was conducted which shows linearity for both monomers after a very short induction period (Figure 10A). This linearity confirmed a constant radical concentration during the copolymerization. MLA was converted quite rapidly in comparison to DMA. Therefore, the copolymerization trend seems to follow a gradient copolymer. This copolymerization process can be also identified in Figure 10B in which the highest value of the dispersity (*Đ* = 1.35) corresponds to a quantitative conversion of MLA but to approximately 20% of the total revenue. After this point, the dispersity reduces until 1.23, corresponding to a dominant DMA part. An evidence for the gradient copolymerization can be found in the ^1^H NMR spectrum by two separate lactide CH signals for the part of MLA and the copolymer part with DMA (Figure S22, Supporting Information File 1). In addition, at low conversion a rapid increase of the molecular weight of *M*_n_ = 4 000 g mol^−1^ (*M*_n theo_ = 1 300 g mol^−1^) can be observed (Figure S23 and Table S8, Supporting Information File 1). This observation has already been described in the literature and termed “hydrid behavior”. It is characterized by a rapid increase in molecular weight in the initial stage due to deviation from the ideal kinetic behavior, leading to a mixed form of free radical and controlled radical polymerization followed by a controlled increase in molecular weight up to high monomer conversions which is responsible for the poor matches to the theoretical *M*_n_ values.

The semi-logarithmic plot of conversion against time of run 13 (ratios 75/25, Table 5) refer to Figure S24 show almost linearity for MLA, but with low conversion compared to the known rapid polymerization behavior. However, from the beginning until 8 h no conversion of DMA was observed, the finally 55% conversion of DMA were achieved afterwards until 22 h. The evolution of *M*_n_ and *Đ* with conversion could not be evaluated due to overlapping signals in the SEC with the solvent DMF. Only at the end of the kinetic at 22 h the copolymer shows a useful value of *M*_n_ = 10 700 g mol^−1^ and *Đ* = 1.6 with incorporated ratio of DMA/MLA of 60/40 determined by ^1^H NMR spectroscopy. The theoretical molecular weight *M*_n theo_ = 14 000 g mol^−1^ is higher than the achieved *M*_n_ which is a sign for the occurrence of transfer reactions. However, in the repetition of the kinetic of run 13 (Table 5) the conversion started with linearity for both monomers after an induction period (refer to Figure S24, Supporting Information File 1) with otherwise the same results (conv. MLA completely and DMA 65%, at 24 h *M*_n_ = 13 400 g mol^−1^ with *Đ* =1.6 (*M*_n theo_ of 17 000 g mol^−1^)).

These findings support the thesis that the copolymerization process of DMA and MLA is based on gradient copolymerization. The low conversion of MLA could be based on a slowly occurring sequence of addition and fragmentation between dormant and active chains because of the radical stabilized by the push–pull substituents. However, with this result it has been shown that the RAFT polymerization is a successful technique for MLA to achieve (co)polymers with narrow dispersities and with almost low molecular weight.

## Conclusion

This first detailed study on the radical polymerization behavior of the cyclic push–pull-type monomer methylenelactide has been conducted. This was performed in comparision to the analogous non-cyclic push–pull-type monomers methyl α-acetoxyacrylate (MAA), ethyl α-acetoxyacrylate, (EAA) and pull-type methyl methacrylate (MMA) and cyclic pull-type α-methylene-δ-valerolactone (MVL).

A deviation from classical free-radical polymerization kinetics was found and correlated with significant self-initiation. A mechanism for the radical formation was proposed and supported by theoretical calculations. With the help of a strongly colored 1,1-diphenyl-2-picrylhydrazyl radical (DPPH) the spontaneous radical formation could also be observed by the naked eye. Furthermore, the copolymerization parameters of MLA with styrene and MMA were obtained and the *Q* and *e* values calculated. The latter allows the prediction of the copolymerization process with further monomers. Finally, this work reports on the first controlled polymerization of methylenelactide and controlled copolymerization with *N*,*N*-dimethylacrylamide via RAFT technique. From the above presented results it can be summarized that MLA represents a highly reactive monomer with a potential for many practical applications and further investigations.

## Supporting Information

Full experimental section containing the description of the materials, characterization methods and syntheses of the obtained polymers, spectroscopic data (^1^H, ^13^C and IR), ^1^H NMR kinetics, UV–vis measurements, polymerization analytics to determine the chain transfer constant, SEC curves of the RAFT initiated (co)polymers, the determination of the copolymerization parameters *Q* and *e* values and force constant.

File 1 Experimental part.
