# Supplementary material for: Methylenelactide: vinyl polymerization and spatial reactivity effects
Source: Beilstein J Org Chem. 2016 Nov 14;12:2378–89. doi: 10.3762/bjoc.12.232 (PMC5238530; doi:10.3762/bjoc.12.232)
Supplement: File 1 — Experimental part. [file Beilstein_J_Org_Chem-12-2378-s001.pdf]

Supporting Information

for

Methylenelactide: vinyl polymerization and spatial reactivity effects

Judita Britner and Helmut Ritter*

Address: Institute of Organic Chemistry and Macromolecular Chemistry, Heinrich-Heine-University, Universitätsstraße 1, 40225 Düsseldorf, Germany

Email: Helmut Ritter* - h.ritter@hhu.de

* Corresponding author

Experimental part

Materials

Methylenelactide (MLA) [1], methyl α-acetoxyacrylate (MAA), ethyl α-acetoxyacrylate (EAA), α-methyl-δ-valerolacton (MVL), 2-(((ethylthio)carbonothioyl)thio)-2-methylpropanoic acid (EMP) and 4-cyano-4-((ethylthio)carbonothioyl)thio)pentanoic acid (CTA) were synthesized according to the literature [2-6].

(3*S*)-*cis*-3,6-Dimethyl-1,4-dioxane-2,5-dione (98%), 2,2′-azobis(2-methylpropionitrile), 4,4'-azobis(4-cyanopentanoic acid), 2-bromoisobutyric acid, *N*,*N*-dimethylacrylamide (DMA), absolute ethanol, ethyl formate (97%), methyl methacrylate (MMA), methyl pyruvate (95%), paraformaldehyde (95%), *S*,*S*-dibenzyl trithiocarbonate (DBTTC) (97%), 2-cyano-2-propyl dodecyl trithiocarbonate (CPDTTC) (97%) and styrene were purchased from Sigma-Aldrich. Benzoyl peroxide (75%), *N*-bromosuccinimide (99%) and ethyl pyruvate (97%) were purchased from Alfa-Aesar. Extra dry *N*,*N*-dimethylformamide (99.8%), ethanethiol (99%) and carbon disulfide were purchased from Acros organics. δ-Valerolactone was received from Evonik Industries, dried over calcium hydride and distilled according to the needs. Acetic anhydride, acetone, diethyl ether, 1,4-dioxane, methylene chloride, methanol, pyridine, triethylamine and tetrahydrofuran were used in p. a. quality. The required amount of 1,4-dioxane and diethyl ether before use was columned over basic alumina to remove peroxides and water. Triethylamine was stirred in potassium hydroxide for 48 h and then distilled and used immediately.

Characterization methods

^{1}H NMR spectra were recorded using a Bruker Avance III 600 spectrometer at 600 MHz or on a Bruker Avance III 300 spectrometer at 300 MHz. The deuterated solvents were used as internal standards. ^{1}H NMR measurements for determination of monomer conversion were measured on a Bruker Avance III 300 spectrometer at 300 MHz. Withdrawn samples of 50 μL

monomer solution at the beginning and at certain points of time and at the end of the polymerization were terminated in liquid nitrogen and diluted with 0.7 mL of DMSO-d_6 including a constant amount of benzene-d_6 as reference. For the determination of the conversion of MLA as an example, the integrals of one vinylic proton (5.81–5.75 ppm) was employed and compared with the reference. In the RAFT initiated copolymerization the integral of one vinylic proton of *N*,*N*-dimethylacrylamide (DMA) (6.19–6.05 ppm) and one vinylic proton of MLA (5.81–5.75 ppm) in comparison with the reference, benzene-d_6, were determined for the conversion. ^{13}C NMR spectra of the polymers were measured as follows: sweep width: 36057.691 Hz, data points: 65536, line broadening: 1 Hz; DEPT-135: sweep width: 24038.461 Hz, data points: 65536, line broadening: 1 Hz; ATP: sweep width: 36057.691 Hz, data points: 65536, line broadening: 3 Hz.

High-performance liquid chromatography (HPLC) measurements were carried out with a BioTek Kontron Instruments construction, type System 525. Detection was performed with a Dioden Array detector at λ = 220, 230 and 270 nm. A CC 250/4 Nucleodur 100-5 C18 ec (Macherey-Nagel, length 250 mm, diameter 4 mm, particle size 5 µm) was used as reverse phase column. A mixture of acetonitrile/water (60/40) was used as eluent with a flow rate of 0.5 mL/min under isocratic conditions. Removal of polymer particles from monomeric molecules was achieved by centrifuge and filtration of the sample and in addition after injection in the HPLC by an upstream LiChroCART®4-4 pre-column. (Merck KGaA). The calibration lines are listed in Figure S13.

Infrared spectra (IR) were measured on a Nicolett 6700 Fourier transform equipped with a diamond single bounce attenuated total reflectance accessory at room temperature. Theoretical calculations were performed with the density functional theory (DFT) by using the software Spartan`16 with the method B3LYP and the basis set 6-31G.

Dynamic differential scanning calorimetry (differential scanning calorimetry, DSC) was performed on a DSC 822e equipped with the samples Sampler TSO801RO of the company

Mettler Toledo. The heating and cooling rates were 10 K/min in the first cycle and 15 K/min in the following (a total of four cycles).

Size exclusion chromatography (SEC) measurements were performed using a ViscotekGPCmax VE2001 system. The System has a column set compromising one MZ-Gel-SDplus, 100 Å pore size and 10 µm particle size, 50 × 8.0 mm [Length × ID]pre-column and two MZ-Gel SDplus linear, 10 µm particle size, 300 × 8.0 mm [Length × ID]columns. The columns were constantly heated to a temperature of 60 °C. *N*,*N*-Dimethylformamide (0.05 M LiBr) was used as eluent at a flow rate of 1 mL min^{-1}. For detection a Viscotek VE 3500 RI detector was used. The system was calibrated with polystyrene standards of a molecular range from 1280 g mol^{-1} to 1373000 g mol^{-1}.

UV–vis spectroscopic measurements were carried out using a dual-trace spectrometer Specord®210 Plus from Analytik Jena AG. Method: Lamp change at 320*, integration time 0.02 s, slit = 1 nm, delta lambda = 1.0, speed = 50 m/s, range 380–800 nm.

Theoretical molecular weights were calculated for the homopolymerization and copolymerization according to the following equations:

$$M_{n,theo} = \text{conv.} \cdot M_{(Monomer)} \cdot \frac{[\text{Monomer}]_0}{[\text{RAFT-agent}]0} + M_{(RAFT\text{-}agent)}$$

$$M_{n,theo} = \text{conv.}_{(M1)} \cdot M_{(M\,1)} \cdot \frac{[[\text{M1}]_0}{[\text{RAFT-agent}]_0}$$

$$+ \text{conv.}_{(M\,2)} \cdot M_{(M\,2)} \cdot \frac{[\text{M2}]_0}{[\text{RAFT-agent}]_0}$$

$$+ M_{(RAFT\text{-}agent)}$$

Verifying of ideal polymerization of MLA

For each sample 2.33 mL from a 1.812 mol L^{-1} stock solution of MLA in 1,4-dioxane was placed in a 50 mL two-necked flask and degassed with nitrogen for 15 min. The variable amount of AIBN (see Table 1) was dissolved with 1 mL of withdrawn monomer solution in an inert atmosphere and afterward backfilled to the two-necked flask. After an additional

degassed time of 10 min the flask was set in an oil bath at 70 °C which was controlled over an internal thermometer. At 1.5 min the polymerization was terminated by adding hydroquinone monomethylether and cooling with liquid nitrogen. The polymer solution was allowed to warm-up to room temperature and pouring into 40 mL of methanol. The precipitated polymer was centrifuged, methanol was decanted off and the polymer was dried at 60 °C in vacuum ($8 \cdot 10^{-2}$ mbar).

DPPH kinetics

In a microwave tube equipped with a stirring bar, MLA (0.515 g, 3.62 mmol) was added and dissolved in 2 mL of a freshly prepared stock solution of DPPH in 1,4-dioxane (c_{DPPH} = 0.28 mM). The mixture was degassed by five freeze-pump-thaw cycles before the tube was backfilled with argon. Under inert conditions the complete solution was inverted into the cuvette by a syringe. The cuvette was placed in the UV–vis spectrometer which was preheated to 70 °C. The disappearance of the absorption at 525 nm of DPPH at 70 °C was followed.

Synthesis of block copolymers

MLA (0.6 g, 4.22 mmol) was added to a two-necked flask dissolved with 4.04 mL 1,4-dioxane and degassed for 20 min. Before the flask was set in an oil bath at 70 °C, a sample of 20 μL was taken. This sample was diluted according to the "Determination of the copolymerization parameter" and measured by HPLC. After 24 h a further sample was taken and measured on the HPLC to detect the MLA residue. Then 1 mL of the polymerization mixture was taken and precipitated in 10 mL of cold methanol. To the remaining polymerization was added one equivalent of styrene (0.326 g, 360 μL, 3.13×10^{-3} mol), diluted in 1.27 mL 1,4-dioxane which had been degassed previously. After further 24 h the polymerization was cooled with liquid nitrogen to stop the polymerization. For determination

of the end concentration of styrene a last sample was taken and the polymer was poured in 50 mL of cold methanol and dried in vacuum (8×10^{-2} mbar) at 60 °C.

General procedure for the RAFT (co)polymerization of MLA

In a microwave tube equipped with a stirring-bar MLA (300 mg. 2.11 mmol, 98.87 equiv) was dissolved in 1,4-dioxane under ice (1.14 ml, 80 wt %.). 21.10 µL of a freshly prepared stock solution containing 1 molar EMP and 0.125 molar AIBN was added quickly to the tube and immediately freeze with liquid nitrogen (21.10 µL of the stock solutions correspond to 4.80 mg (0.0211 mmol, 1 eq.) of EMP and to 0.43 mg (2.64×10^{-3} mmol, 0.125 equiv) of AIBN). The reaction mixture was degassed by five freeze-pump-thaws. A NMR sample (50 µL) was withdrawn for the determination of the MLA conversion and dissolved in 0.7 mL DMSO-d_6 including benzene-d_6 as reference cycles during the tube was backfilled with argon. After 18 h in an oil bath at 70 °C, controlled over an internal thermometer of a second tube, the tube was cooled with liquid nitrogen to stop the polymerization. The polymer solution was allowed to warm-up to room temperature. An NMR sample (50 µL) was withdrawn for the determination of the MLA conversion. A conversion of >99% was calculated based on the NMR data (see "Characterization methods" for details of the calculation). The polymer solution was diluted with 1 mL of acetone, poured in 50 mL of cold methanol, filtered and dried in vacuum ($8 \cdot 10^{-2}$ mbar) at 60 °C to yield the polymer as pale yellow solid. (190 mg, 64%, M_{n_theo} = 14 302 g mol^{-1} , SEC(DMF):M_{n_SEC} = 42 959 g mol^{-1}, $Đ$ = 1.6).

In case of the copolymerization of MLA with *N*,*N*-dimethylacrylamide (DMA) the polymer solution was diluted in 1 mL of acetone and poured in 50 mL of diethyl ether, filtered, and dried in vacuum (8×10^{-2} mbar) at 60 °C. This purification procedure was repeated once.

Determination of the copolymerization parameter

The residual monomer content of six different polymerization mixtures (refer to Table S5, S7) was determined by high-performance liquid chromatography (HPLC), respectively. The conversion was kept below 27%.

The implementation is described by the example of run 18-1 (Table S3).

0.104 mL of a freshly prepared stock solutions of 1.7 molar MLA in 1,4-dioxane, 0.192 mL of styrene and 0.655 mL of 1,4-dioxane were added in a 25 mL two-necked flask and degassed with nitrogen for 15 min. Then 18.6 µL of a freshly prepared stock solutions of 1 mol AIBN was added under nitrogen and the mixture was degassed for further 5 min. Before the flask was set in an oil bath at 70 °C for 12 minutes, three samples of 20 µL were withdrawn. The withdrawn samples were diluted with 2 mL methanol respectively and centrifugalized for 1.5 minutes. Then the samples were filtrated and 0.5 mL of the sample was diluted with 1.25 mL acetonitrile and 1 mL methanol. The samples were injected to the HPLC (see "characterization methods") and the average peak area of the three measurements was taken as start concentration (peak area MLA at 4.43–5.50 min and styrene at 10.60–12.50 min). After the induced end of the polymerization at 12 min the polymerization was cooled down by liquid nitrogen. According to the beginning the monomer concentrations were determinate through the average results of three withdrawn samples.

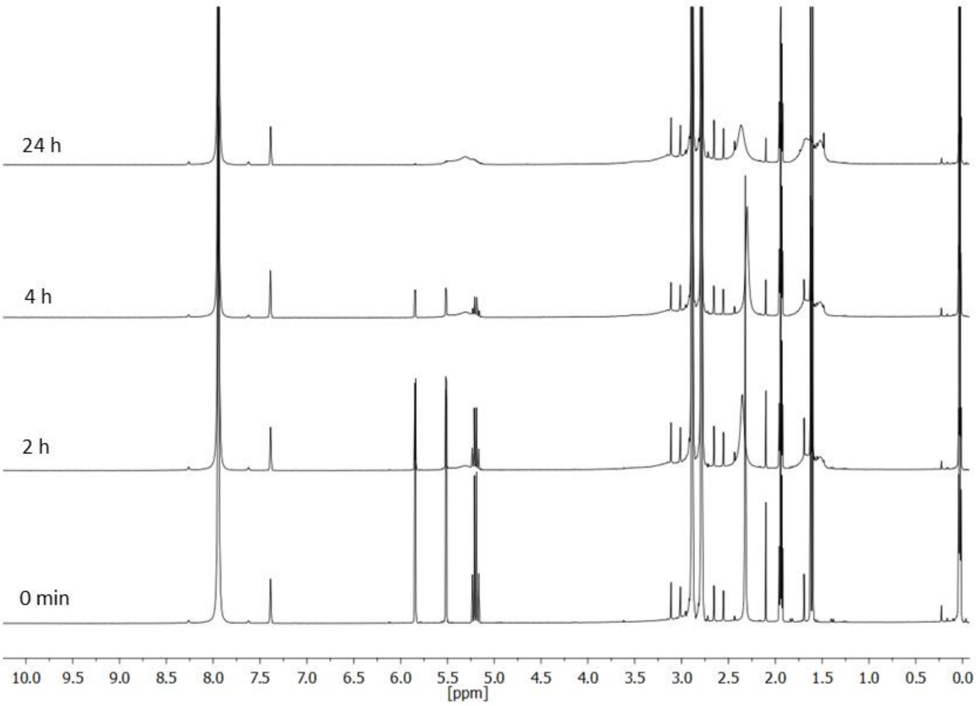

Figure S1: ^{1}H NMR spectra at 0 min, 2 h, 4 h and 24 h from the AIBN initiated free-radical polymerization of MLA in dry DMF without ring-opening or hydrolysis of MLA (c_{MLA} = 1.6 mol L^{-1}, 80 wt % DMF; 70 °C).

Table S1: Overview of the spectroscopic data's of the double bond of the push-pull monomers. Dipole moment calculation was done over Spartan with the density functional theory (DFT).

		^{1}H NMR		^{13}C NMR		dipole moment	IR
		$H_2C=C$		$H_2C=C$	$H_2C=C$	$H_2C=C$	$H_2C=C$
		H_{cis} [ppm]	H_{trans} [ppm]	[ppm]	[ppm]	[Debye]	[cm^{-1}]
MLA (1)	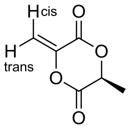	5.77	5.56	143.69	108.31	2.09	1645.80
MMA	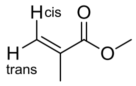	6.03	5.66	135.77	125.59	4.10	1638.10
MAA	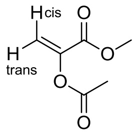	6.02	5.65	144.04	114.67	3.79	1649.00
EAA	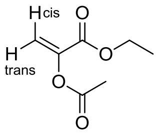	5.99	5.62	144.31	114.32	2.26	1649.50
MVL	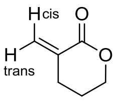	6.22	5.41	134.09	127.74	4.65	1625.40

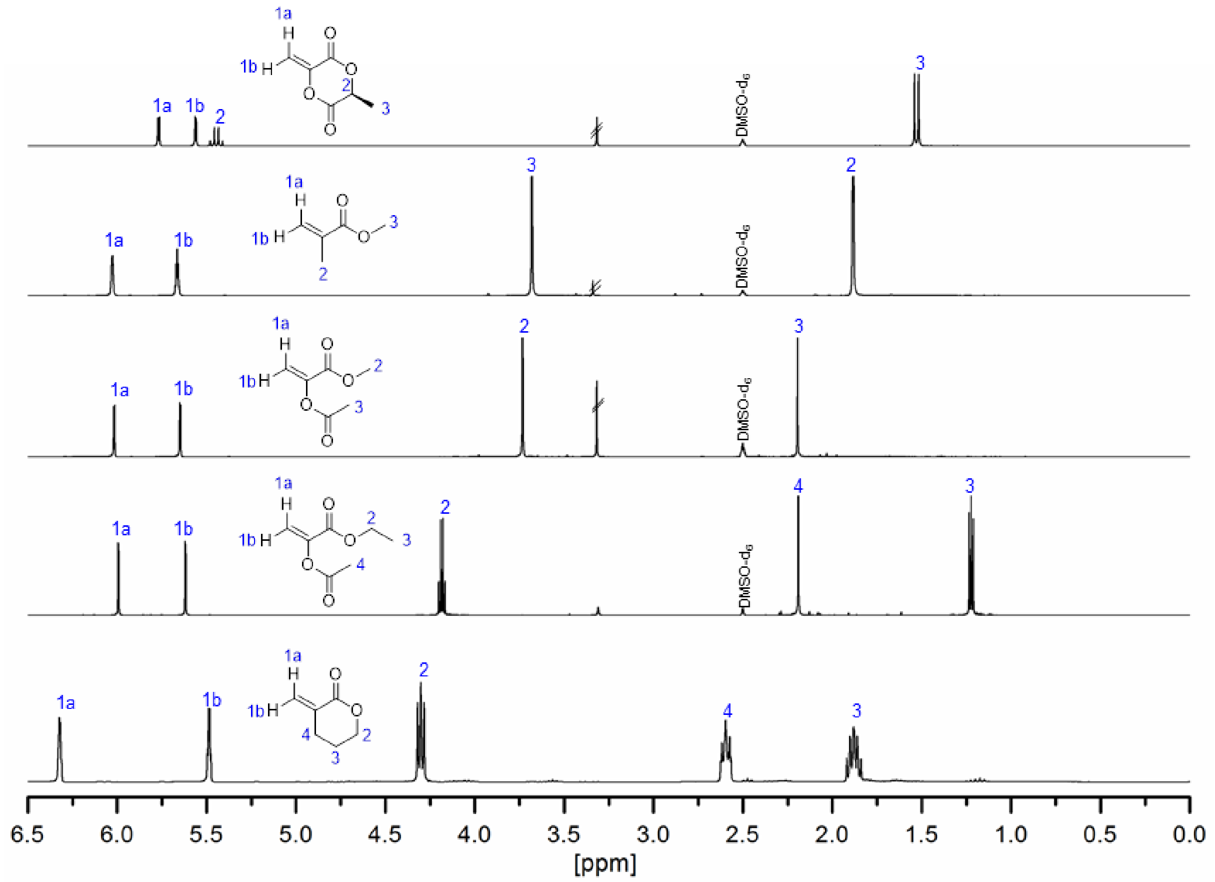

Figure S2: ^{1}H NMR spectra of MLA, MMA, MAA, EAA in DMSO-d_6 (300 MHz) and MVL in $CDCl_3$ (300 MHz).

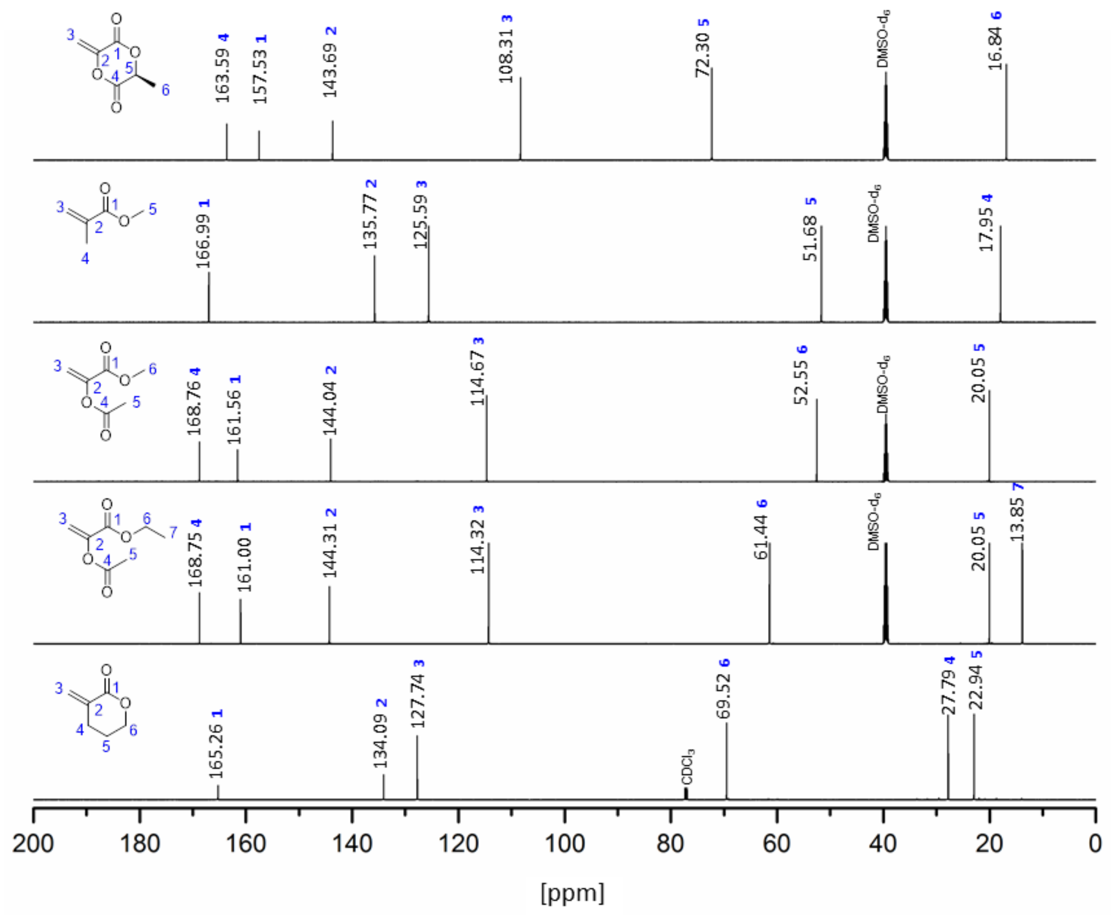

Figure S3: ^{13}C NMR spectra of MLA, MMA, MAA, EAA in in DMSO-d_6 and MVL in CDCI$_3$ (150 MHz).

Table S2. Determined analytics for the polymer s of MLA, MMA, MAA, EAA and MVL (80 wt % DMF, 1 mol % AIBN, 70 °C and 24 h).

**polymer**	$\boldsymbol{M_n}$ a) [g mol^{-1}]	$\boldsymbol{Đ}$ a)	$\boldsymbol{T_g}$ b) [°C]
poly(MLA)	74 900	2.3	249
poly(MMA)	23 700	1.8	105
poly(MAA)	21 600	1.5	143
poly(EAA)	31 600	1.7	108
poly(MVL)	584 300 & 20 400	1.6 & 1.3	186

a) SEC: Determined by PS-calibrated SEC (solvent DMF, 0.05M LiBr)

b) DSC: average of the second and third heating cycle (15 ° C min-1)

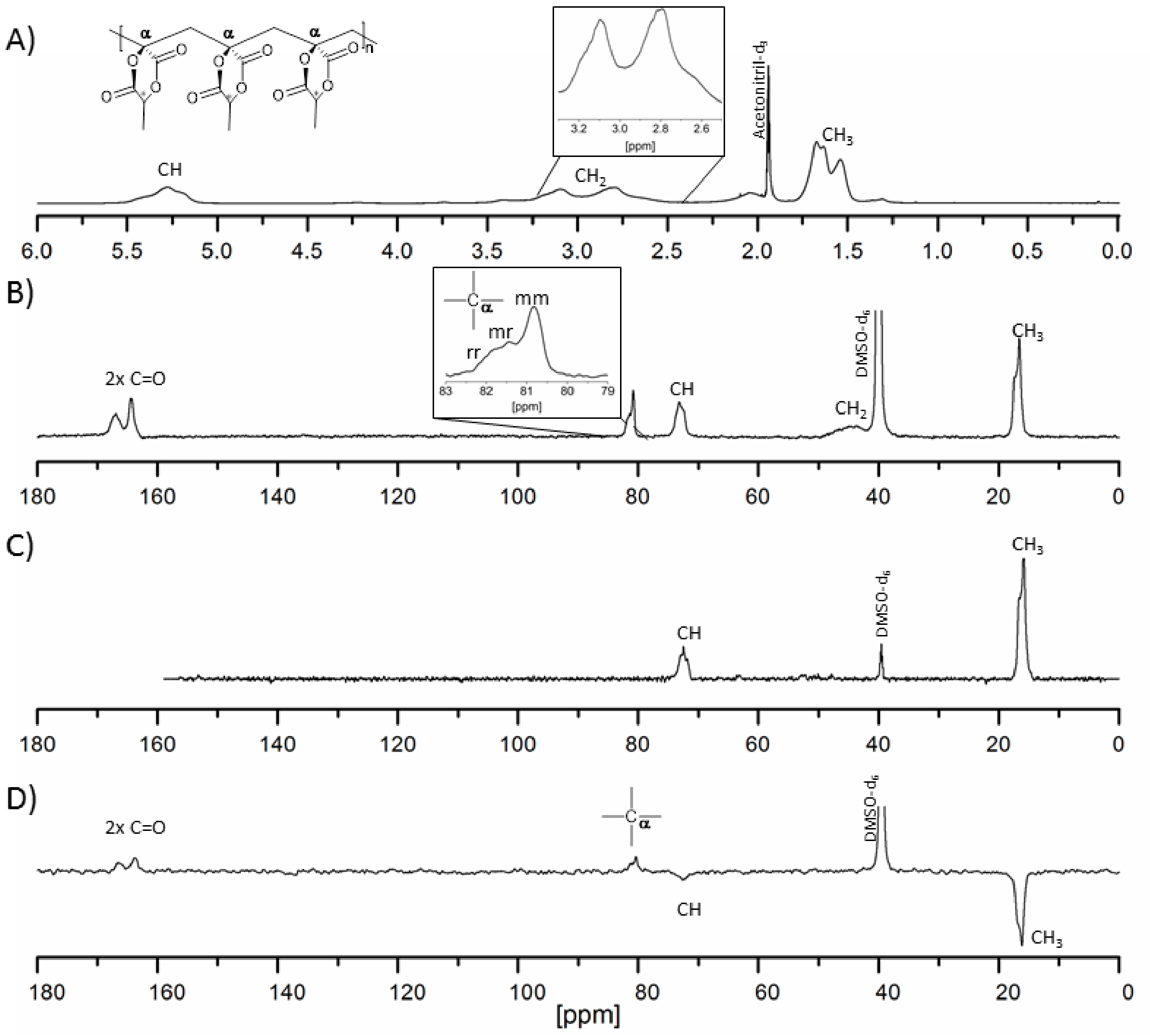

Figure S4: Spectra of poly(MLA): A) ^{1}H NMR (300 MHz, CD_3CN, rt, c = 5 mg·mL^{-1}); B) ^{13}C{H} NMR, C) DEPT-135, D) ATP (for B,C and D; 150 MHz, DMSO-d_6, 70 °C , c = 100 mg·mL^{-1})

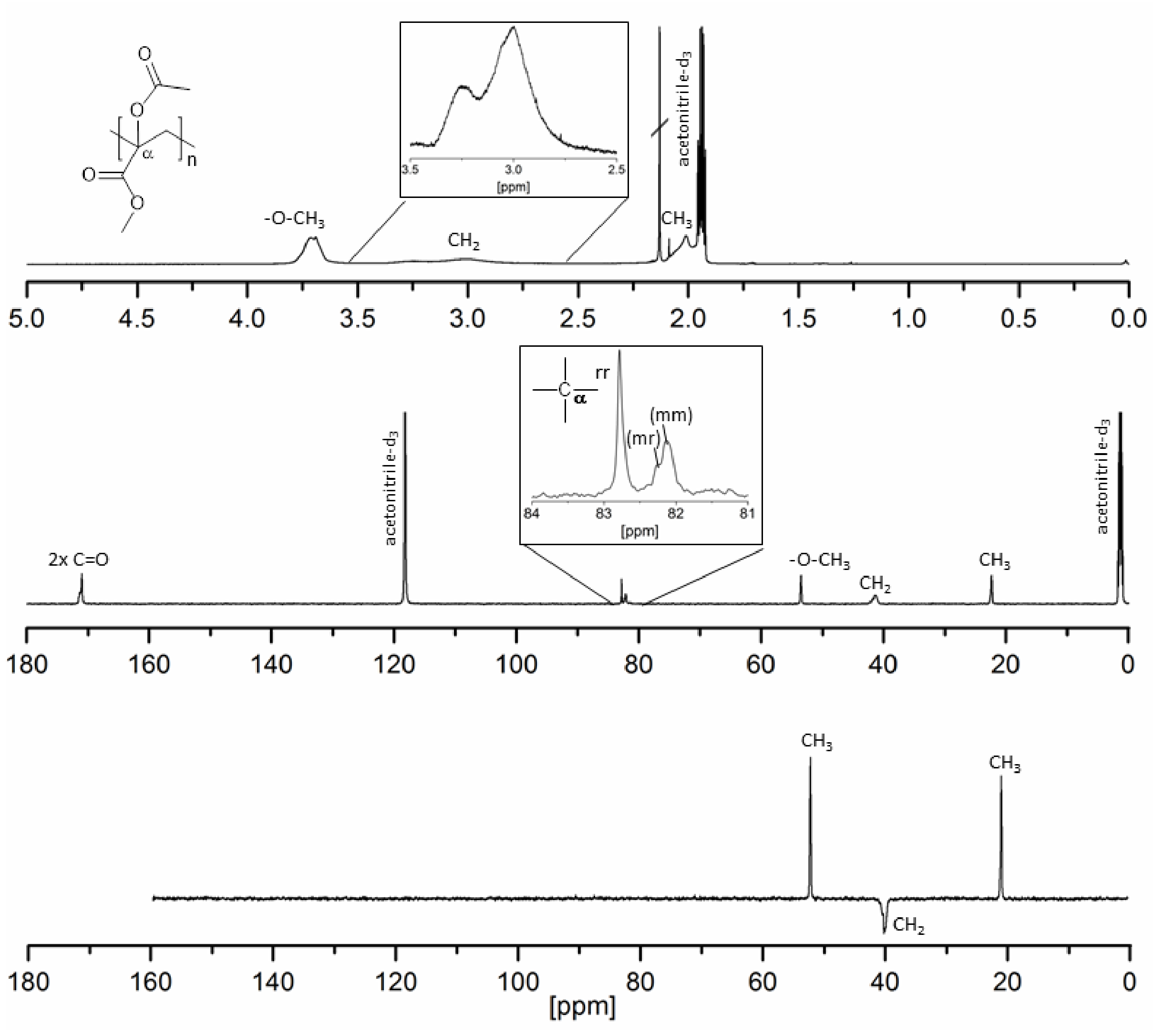

Figure S5: Spectra of poly(MAA): A) ^{1}H NMR (300 MHz, CD_3CN, rt, c = 5 mg·mL^{-1}); B) $^{13}C\{H\}$ NMR, C) DEPT-135 (for B and C; 150 MHz, CD_3CN, 70 °C , c = 100 mg·mL^{-1}).

Found $\nu = 2948.8\ \text{cm}^{-1}$

$\lambda = \frac{1}{\nu} = 3.39 \cdot 10^{-4}\ \text{cm} = 3.39 \cdot 10^{-6}\ \text{m}$

$\nu = \frac{c}{\lambda} = 2.99792558 \cdot 10^{8}\ \frac{\text{m}}{\text{s}} / 3.39 \cdot 10^{-6}\ \text{m} = 8.84 \cdot 10^{13}\ \frac{1}{\text{s}}$

$\omega = 2\ \pi\ \nu = 5.5545 \cdot 10^{14}\ \frac{1}{\text{s}}$

m_{eff} = reduced mass for CH = $1.5342 \cdot 10^{-27}$ kg

$\boxed{k = m_{eff} \cdot \omega^2}$ $\rightarrow k = 473.34\ \frac{\text{kg}}{\text{s}^2} = 473.34\ \frac{\text{N}}{\text{m}}$

Figure S6: Calculating method intended the force constant.

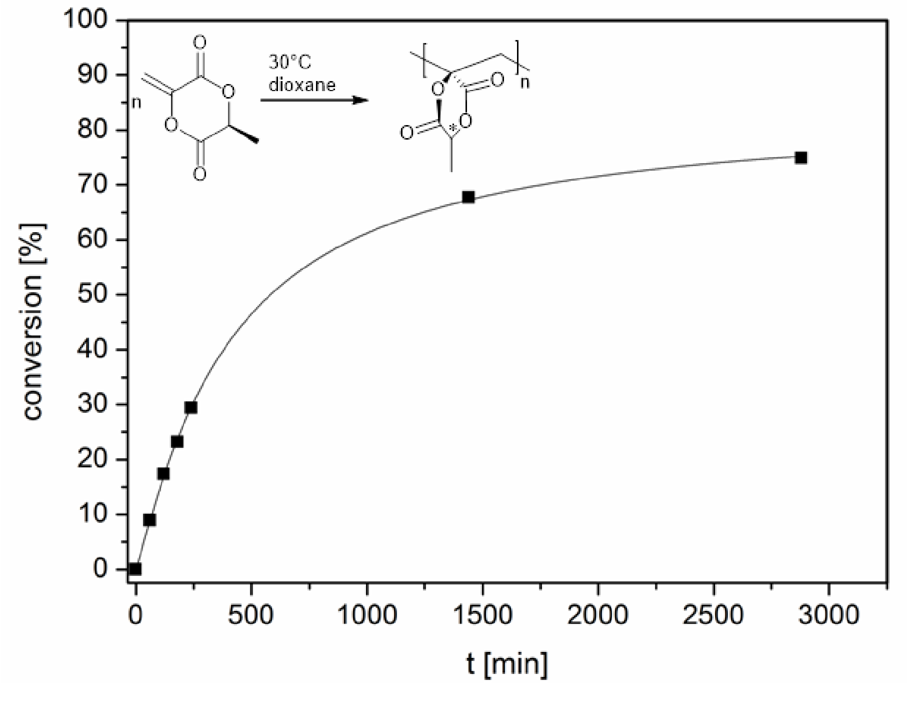

Figure S7: Conversion plot of the free-radical polymerization of MLA in 1,4-dioxan at 30 °C DMF without AIBN (c_{MLA} = 1.8 mol L^{-1}, 30 °C).

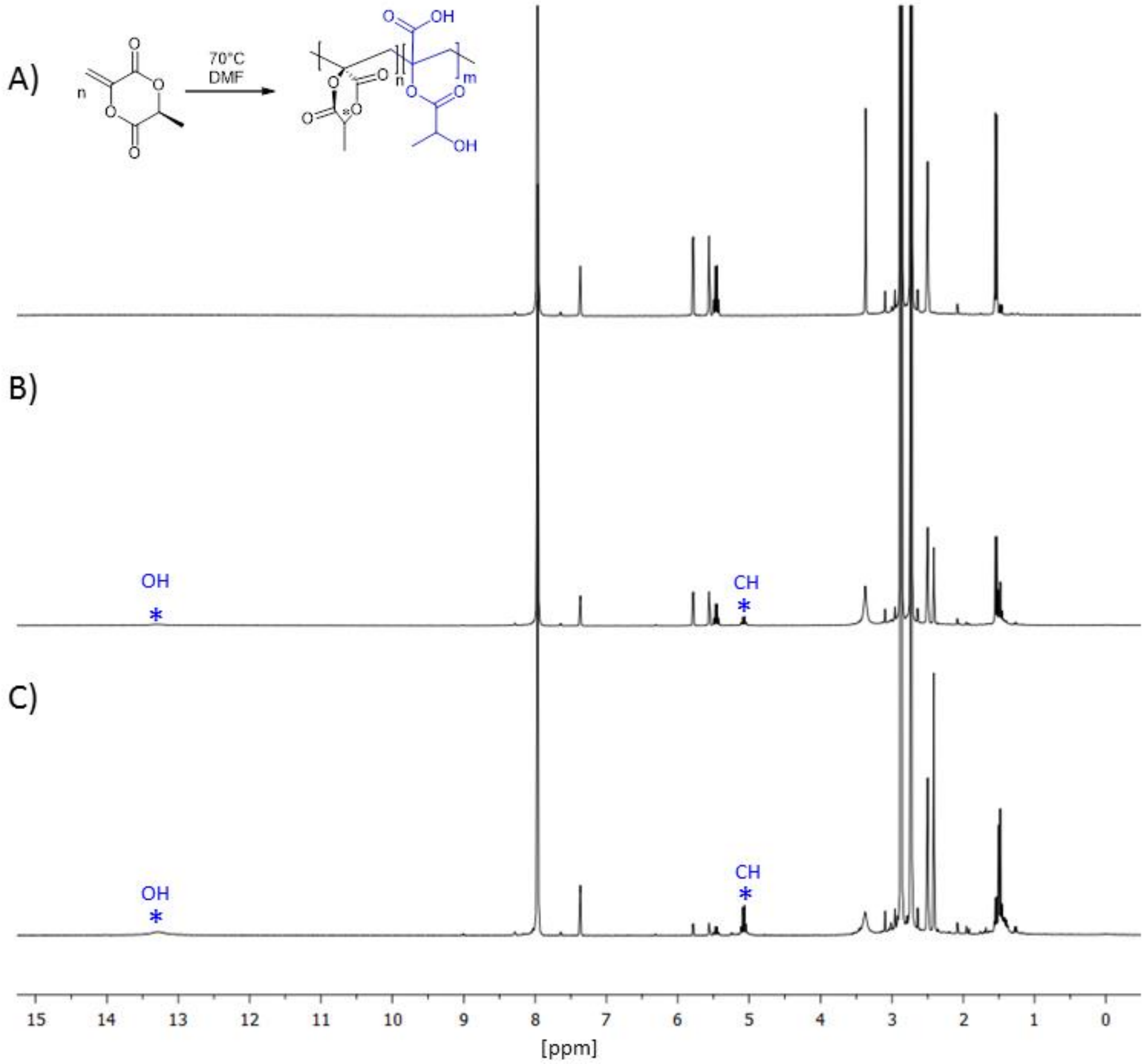

Figure S8: ^{1}H NMR spectra at A) 0 min, B) 8 h and C) 28 h from the self-initiated free-radical polymerization of MLA in DMF. *denotes the opened part (c_{MLA} = 1.6 mol L^{-1}, 70 °C).

[DPPH·]	nm	absorption
0.28	519	2.8718
0.25	517	2.5102
0.2	518	2.07527
0.15	517	1.56561
0.1	517	1.02965

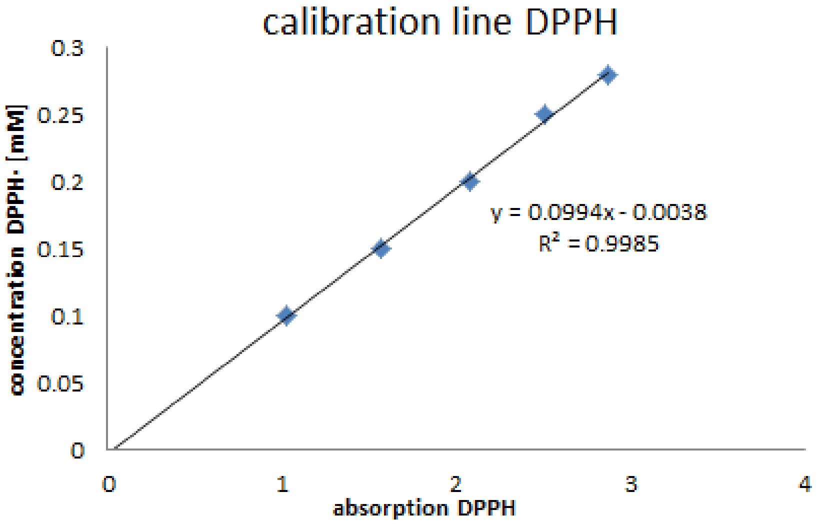

Figure S9: Calibration line of DPPH in the range of 0.1 to 0.28 mM in 1,4-dioxane.

t [s]	absorption	DPPH· [mM]
0	2.3644	0.2312
5	2.2659	0.2214
10	2.2500	0.2199
20	2.2269	0.2176
40	2.1843	0.2133
80	1.9936	0.1944
240	1.6982	0.1650
300	1.5159	0.1469
900	0.1097	0.0071

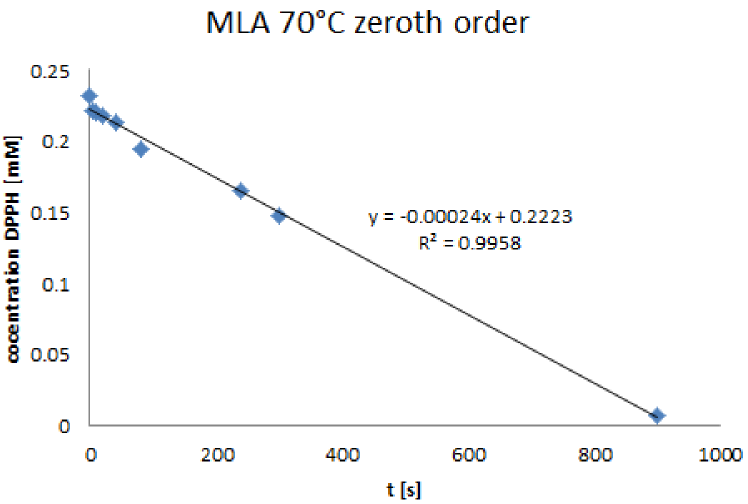

Figure S10: UV–vis data of the disappearance of DPPH in the self-initiated polymerization of MLA at 70 °C with the plotted DPPH concentration against time (c_{MLA} = 1.8 mol L^{-1} in 1,4-dioxane rate constant $k = 2.4 \times 10^{-4}$ mM s^{-1}).

t [s]	absorption	DPPH· [mM]
0	2.2357	0.2184
10	2.2109	0.2160
20	2.1945	0.2143
60	2.1367	0.2086
120	2.0778	0.2027
180	2.0260	0.1976
240	1.9803	0.1930
300	1.9376	0.1888
360	1.9033	0.1854
1080	1.8227	0.1774
1140	1.7781	0.1729
1440	1.6844	0.1636
2400	1.3130	0.1267
2880	0.8487	0.0806
3720	0.3433	0.0303

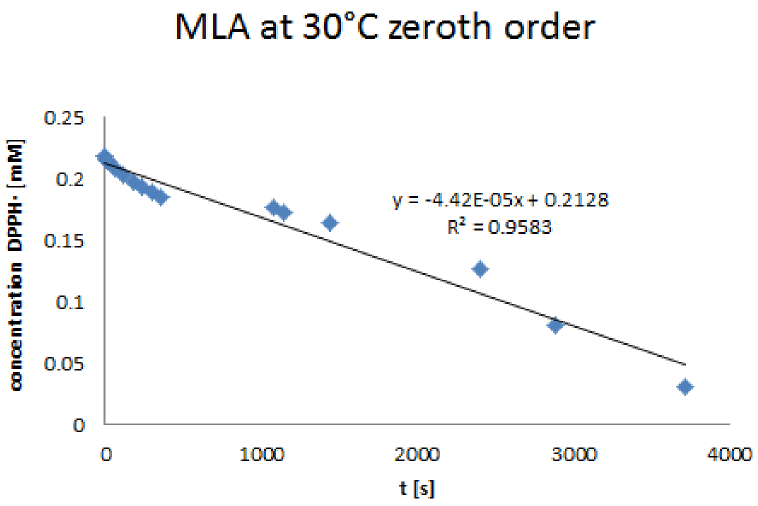

Figure S11: UV–vis data of the disappearance of DPPH in the self-initiated polymerization of MLA at 30 °C with the plotted DPPH concentration against time. (c_{MLA} = 1.8 mol L^{-1} in 1,4-dioxane, rate constant k = 4.42 × 10^{-5} mM s^{-1}).

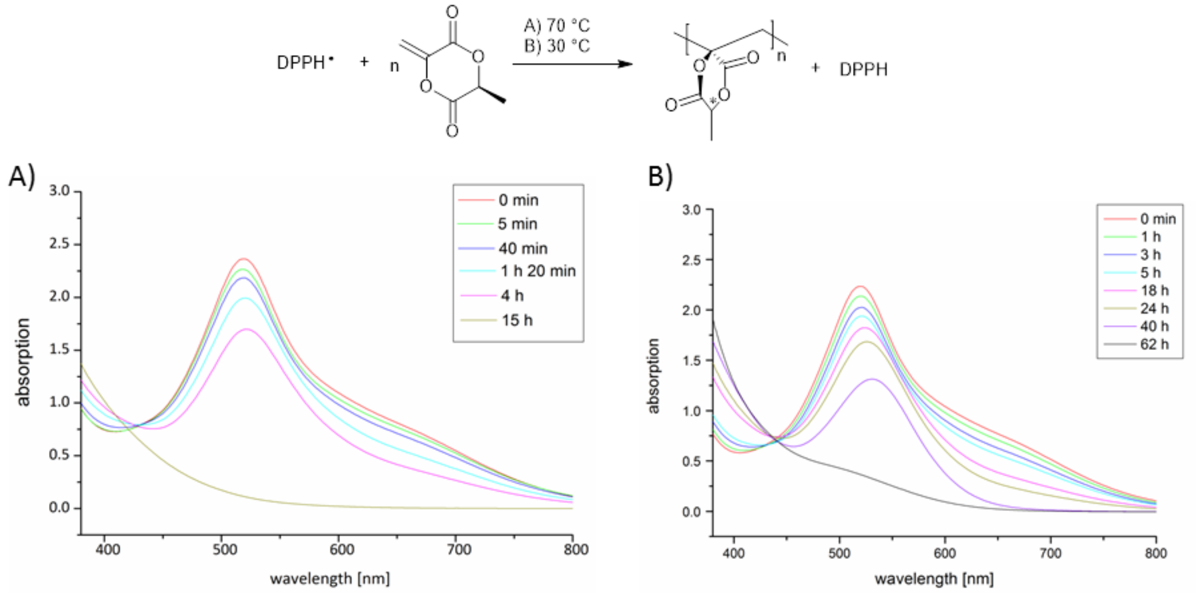

Figure S12: Disappearance of DHHP at the self-initiated polymerization of MLA at A) 70 °C and B) 30 °C (c_{DPPH} = 0.28 mM, c_{MLA} = 1.8 mol L^{-1} in 1,4-dioxane).

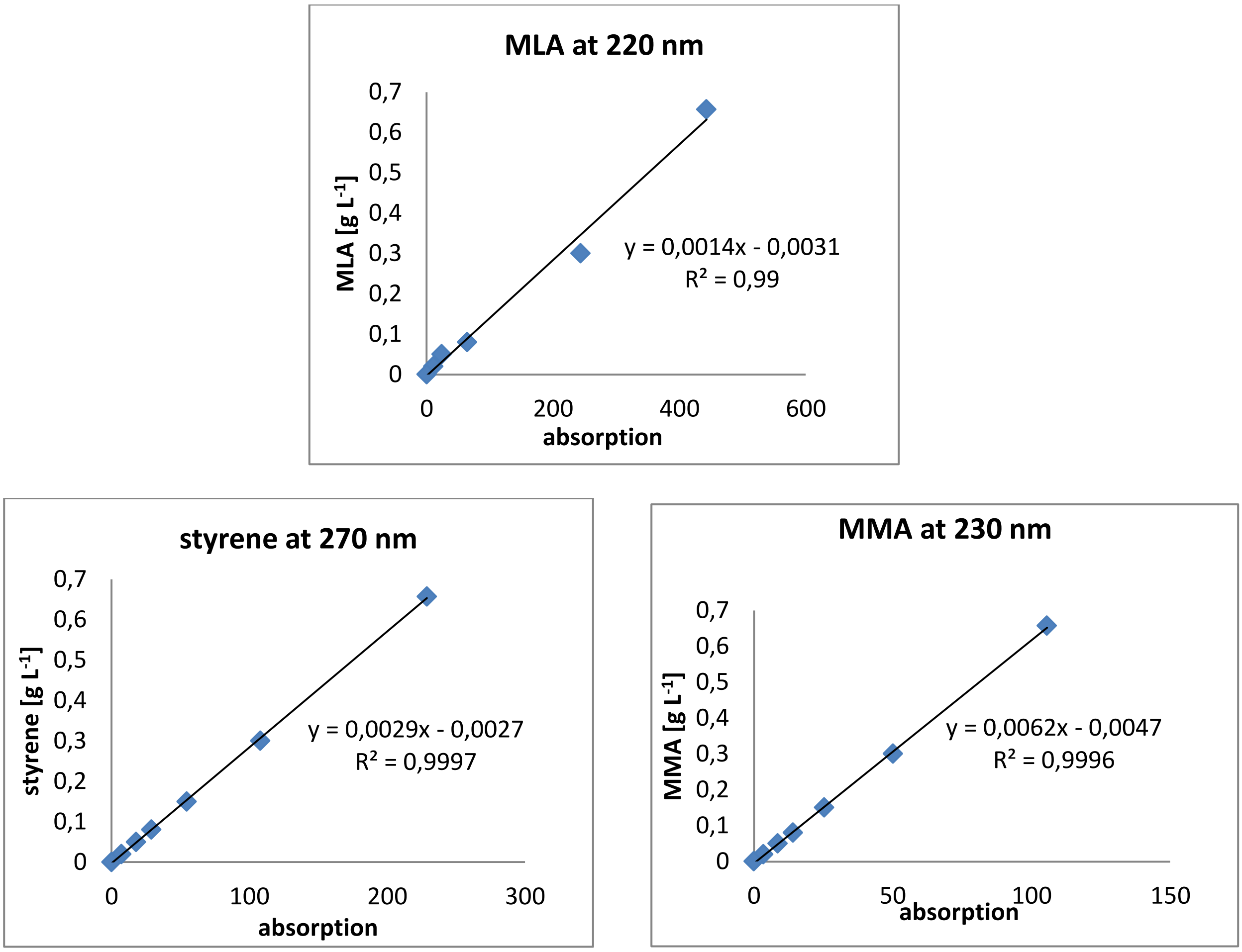

Figure S13: Calibration lines of MLA, styrene and MMA for the HPLC determined copolymerization parameters (eluent: acetonitrile/water 60/40, flow rate 0.5 mL min^{-1}, all concentration samples were measured three times and the average values were used).

Table S3: Different polymerization mixture for the determination of the copolymerization parameter of the system, MLA and styrene (1 mol % AIBN, 80 wt % 1,4-dioxane, 70 °C).

run	m MLA [mg]	m styrene [mg]	m AIBN [mg]	V dioxane [mL]
18-1	0.025	0.175	3.05E-03	0.78
18-2	0.075	0.125	2.84E-03	0.78
18-3	0.1	0.1	2.73E-03	0.78
18-4	0.125	0.075	2.63E-03	0.78
18-5	0.15	0.05	2.52E-03	0.78
18-6	0.175	0.025	2.42E-03	0.78

Table S4: Copolymerization of MLA and styrene. Analysis data via HPLC of taken samples at the beginning A) and from the induced end at 12 min B) (sample 20 µL diluted with 4.25 mL of a mixture of methanol/acetonitrile). The peak area (at MLA at 4.43–5.50 min and styrene at 10.60–12.50 min) show the average of three measurements.

A)

run **0 min**	peak area MLA=$[M_1]$	$[M_1]_0$ [g/L]	$[M_1]_0$ [mol/L]	peak area Styrene=$[M_2]$	$[M_2]_0$ [g/L]	$[M_2]_0$ [mol/L]	**a** $[M1]_0/[M2]_0$
18-1	17.098	0.021	1.47E-04	193.736	0.559	5.37E-03	0.027
18-2	75.588	0.103	7.23E-04	151.483	0.437	4.19E-03	0.172
18-3	146.784	0.202	1.42E-03	138.160	0.398	3.82E-03	0.373
18-4	180.159	0.249	1.75E-03	102.912	0.296	2.84E-03	0.617
18-5	230.857	0.320	2.25E-03	66.394	0.190	1.82E-03	1.236
18-6	291.249	0.405	2.85E-03	34.201	0.096	9.26E-04	3.073

B)

run **12 min**	peak area $[M_1]$	$[M_1]_{12}$ [g/L]	$[M_1]_{12}$ [mol/L]	$d[M_1]$ [mol/L]	peak area styrene$[M_2]$	$[M_2]_{12}$ [g/L]	Styrol $[M_2]_{12}$ [mol/L]	$d[M_2]$	**b** $d[M_1]/d[M_2]$
18-1	16.029	0.019	1.36E-04	1.05E-05	184.269	0.532	5.10E-03	2.64E-04	0.040
18-2	71.594	0.097	6.83E-04	3.93E-05	150.770	0.435	4.17E-03	1.99E-05	1.981
18-3	141.742	0.195	1.37E-03	4.97E-05	130.273	0.375	3.60E-03	2.20E-04	0.226
18-4	133.507	0.184	1.29E-03	4.60E-04	79.200	0.227	2.18E-03	6.60E-04	0.696
18-5	212.951	0.295	2.08E-03	1.76E-04	58.898	0.168	1.61E-03	2.09E-04	0.845
18-6	237.826	0.330	2.32E-03	5.26E-04	24.771	0.069	6.64E-04	2.63E-04	2.004

$\alpha = \sqrt{F\,min \cdot F\,max} = 0.296$

run	F	G	$\frac{G}{(\alpha+F)}$	$\frac{F}{(\alpha+F)}$
18-1	0.019	0.656	2.08	0,06
(18-2	0.015	-0.085	-0.27	0,05)
(18-3	0.614	1.275	1.40	0,67)
18-4	0.547	0.270	0.32	0,65
18-5	1.807	0.226	0,11	0,86
18-6	4.713	-1.540	-0,31	0,94

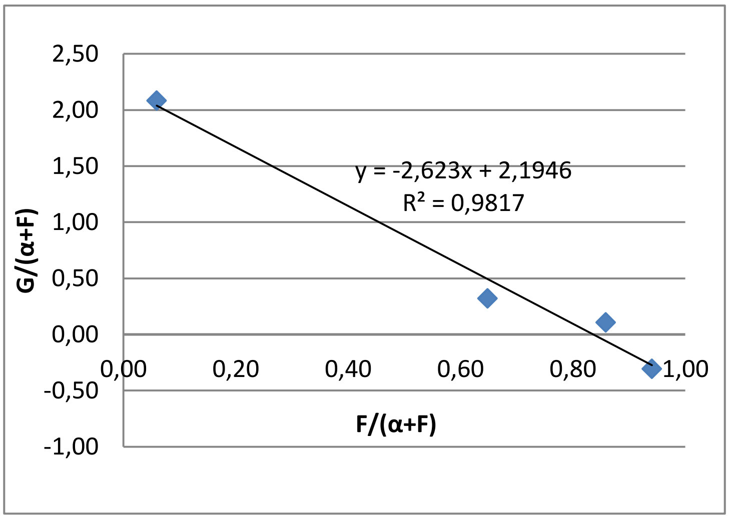

Figure S14: Plot according to *Kelen* and *Tüdös* for the determination of the copolymerization parameters of MLA monomer 1 $[M_1]$ and styrene monomer 2 $[M_2]$.

Table S5: Different polymerization mixture for the determination of the copolymerization parameter of the system MLA and MMA (1 mol % AIBN, 80 wt % 1,4-dioxane, 70 °C).

run	m MLA [mg]	m styrene [mg]	m AIBN [mg]	V dioxane [mL]
19-1	0.025	0.175	3.16E-03	0.78
19-2	0.075	0.125	2.92E-03	0.78
19-3	0.1	0.1	2.80E-03	0.78
19-4	0.125	0.075	2.67E-03	0.78
19-5	0.15	0.05	2.55E-03	0.78
19-6	0.175	0.025	2.43E-03	0.78

Table S6: Copolymerization of MLA and MMA. Analysis data via HPLC of taken samples at the beginning A) and from the induced end at 10 min B) (sample 20 µL diluted with 4.25 mL of a mixture of methanol/acetonitrile/water).The peak area (at MLA 4.43–5.50 min and MMA at 6.11–7.16 min) show the average of three measurements.

A)

run **0 min**	peak area MLA=$[M_1]$	$[M_1]_0$ [g/L]	$[M_1]_0$ [mol/L]	peak area MMA= $[M_2]$	$[M_2]_0$ [g/L]	$[M_2]_0$ [mol/L]	**a** $[M_1]/[M_2]$
19-1	25.05	0.03	2.25E-04	82.49	0.51	5.06E-03	4.45E-02
19-2	75.08	0.10	7.18E-04	62.27	0.38	3.81E-03	1.88E-01
19-3	110.60	0.15	1.07E-03	54.28	0.33	3.31E-03	3.22E-01
19-4	139.33	0.19	1.35E-03	38.94	0.24	2.36E-03	5.71E-01
19-5	192.38	0.27	1.87E-03	28.35	0.17	1.71E-03	1.10E+00
19-6	217.91	0.30	2.12E-03	13.78	0.08	8.07E-04	2.63E+00

B)

run **10 min**	peak area $[M_1]$	$[M_1]_{10}$ [g/L]	$[M_1]_{10}$ [mol/L]	$d[M_1]$ [mol/L]	peak area MMA= $[M_2]$	$[M_2]_{10}$ [g/L]	$[M_2]_0$ [mol/L]	$d[M_2]$ [mol/L]	**b** $d[M_1]/d[M_2]$
19-1	24.59	0.03	2.20E-04	4.56E-06	75.26	0.46	4.61E-03	4.48E-04	1.02E-02
19-2	68.15	0.09	6.50E-04	6.83E-05	56.48	0.35	3.45E-03	3.59E-04	1.90E-01
19-3	100.04	0.14	9.64E-04	1.04E-04	46.35	0.28	2.82E-03	4.92E-04	2.12E-01
19-4	124.85	0.17	1.21E-03	1.43E-04	34.76	0.21	2.11E-03	2.59E-04	5.50E-01
19-5	157.52	0.22	1.53E-03	3.43E-04	23.04	0.14	1.38E-03	3.29E-04	1.04E+00
19-6	165.34	0.23	1.61E-03	5.18E-04	11.25	0.07	6.50E-04	1.57E-04	3.30E+00

$\alpha = \sqrt{F\,min \cdot F\,max} = 0.638$

run	F	G	$\frac{G}{(\alpha+F)}$	$\frac{F}{(\alpha+F)}$
(19-1	0.194	4.316	5.19	0.23)
19-2	0.187	0.802	0.97	0.23
(19-3	0.490	1.199	1.06	0.43)
19-4	0.593	0.467	0.38	0.48
19-5	1.152	-0.045	-0.03	0.64
19-6	2.103	-1.836	-0.67	0.77

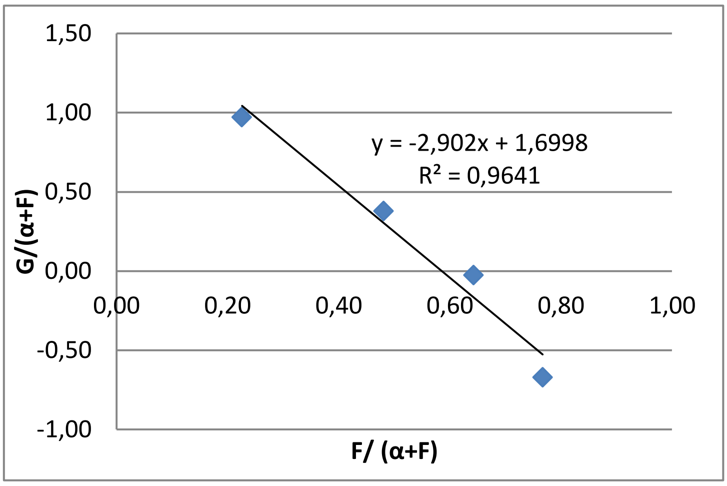

Figure S15: Plot according to *Kelen* and *Tüdös* for the determination of the copolymerization parameter of MLA monomer 1 [M_1] and MMA monomer 2 [M_2].

The Q-e-equation: $r_1 = \frac{k11}{k12} = \frac{Q1}{Q2} \cdot e^{[-e1(e1-e2)]}$, $r_2 = \frac{k22}{k21} = \frac{Q2}{Q1} \cdot e^{[-e2(e2-e1)]}$

⇨ $r_1 \cdot r_2 = e^{[-e1(e1-e2)]} \cdot e^{[-e2(e2-e1)]}$

⇨ $r_1 \cdot r_2 = e^{-(e1-e2)^2}$

⇨ $\sqrt{-[\ln(r_1 \cdot r_2)]} - 0.8 = e_2$

Figure S16: "Q, e" equation by Alfrey–Price reconverted to e_2.

HOEtSH [mmol]	[HOEtSH]/ [MLA]	Mn [g/mol]	Đ	1/Pn
0	0	75 994	3.4	0.0018
(0.0281	0.02	61 151	2.9	0.0023)
0.0352	0.025	41 206	2.4	0.0034
0.0464	0.033	35 373	3.3	0.0040
0.0704	0.05	29 205	3.2	0.0048

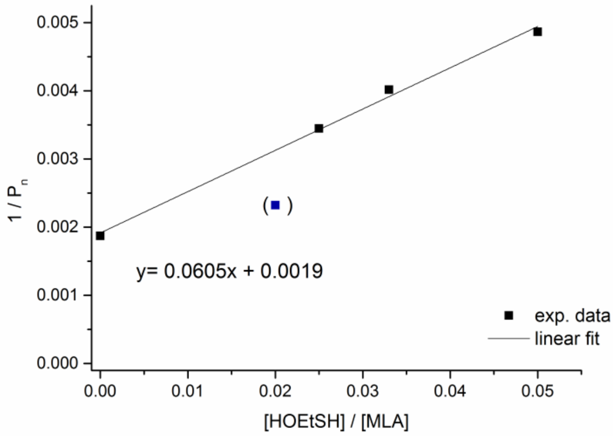

Figure S17: Polymerization approaches with determined analytics and graphical plot to determine the chain transfer constant (C_{tr}) with C_{tr} = 0.06 of the radical polymerization of MLA with mercaptoethanol (HOEtSH) indicate no chain-transfer reaction thus C_{tr} is under 1 (80 wt % 1,4-dioxane, 1 mol %, 70 °C AIBN, 24 h).

Table S7: Polymerization approaches with determined analytics to determine the chain transfer constant of the radical polymerization of MLA with isopropanol (IPA) (C_{tr} = 0.024 (IPA)) and 1-adamantanthiol (1-DCSH) (C_{tr} = 0.06 (1-DCSH) (80 wt % 1,4-dioxane, 1 mol % AIBN, 70 °C, 24 h).

IPA [mmol]	[IPA]/ [MLA]	M_n [g/mol]	Đ	1/Pn
0	0	83 627	4.1	0.0017
0.0281	0.02	72 842	4.1	0.0020
0.0352	0.025	61 591	4.4	0.0023
0.0464	0.033	48 523	3.8	0.0029
(0.0704	0.05	61 221	4.9	0.0023)

1-DCSH [mmol]	[1-DCSH]/ [MLA]	M_n [g/mol]	Đ	1/Pn
0	0	118 210	3.6	0.0012
0.0281	0.02	67 988	3.2	0.0021
0.0352	0.025	49 940	2.6	0.0028
0.0464	0.033	44 336	3.7	0.0032
0.0704	0.05	34 444	3.0	0.0041

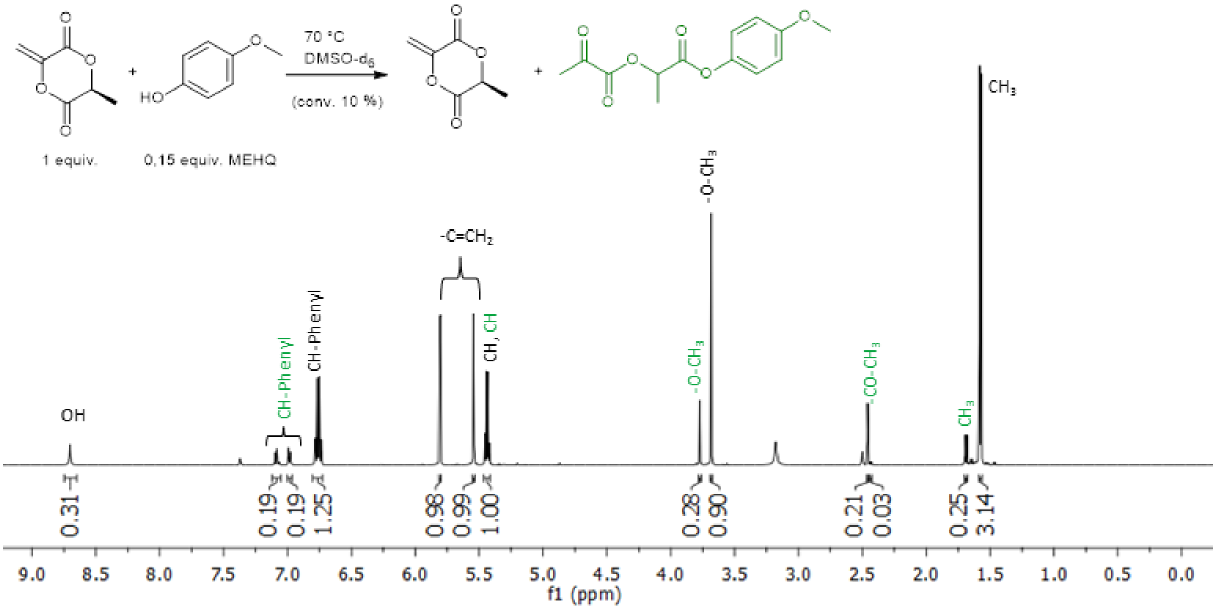

Figure S18: ^{1}H NMR of MLA with 0.15 equivalents of p-methoxyphenol (MEHQ) as inhibitor (600 MHz, DMSO-d_6, 70 °C, $c_{\mathrm{MLA}} = 0.5$ mol·L^{-1})

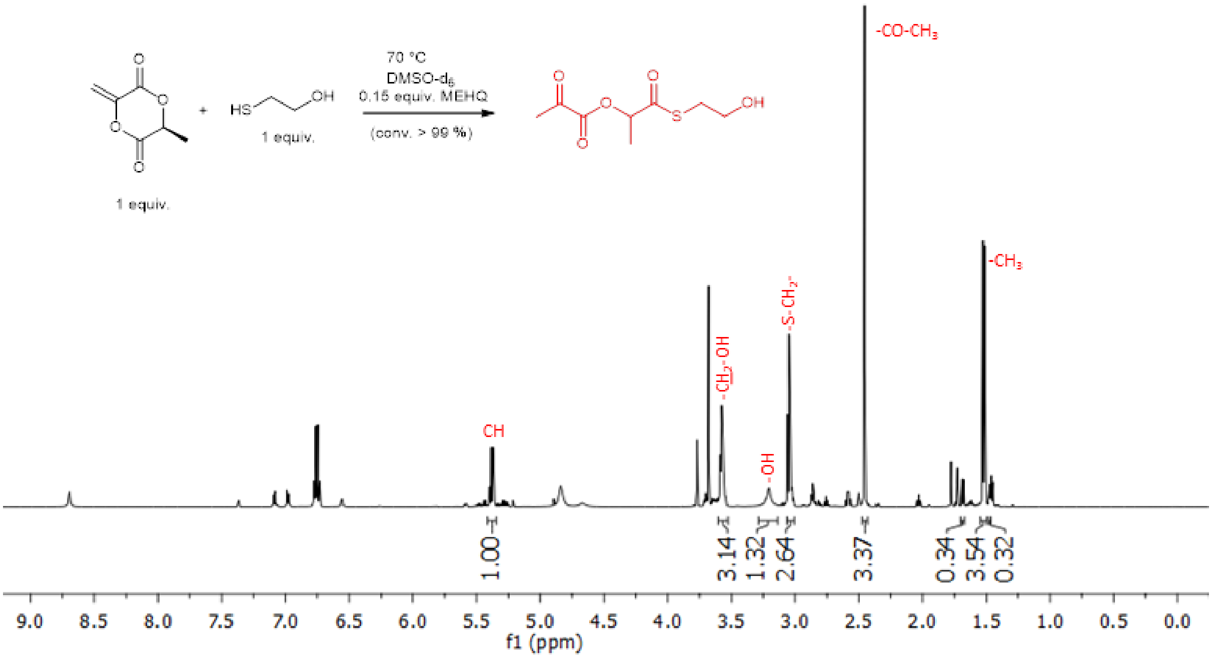

Figure S19: ^{1}H NMR of MLA with 1 equiv of 2-mercaptoethanol and 0.15 equiv of *p*-methoxyphenol (MEHQ) as inhibitor measured after 30 min (600 MHz, DMSO-d_6, 70 °C, $c_{\mathrm{MLA}} = c_{\mathrm{HOEtSH}}$ 0.5 mol·L^{-1}).

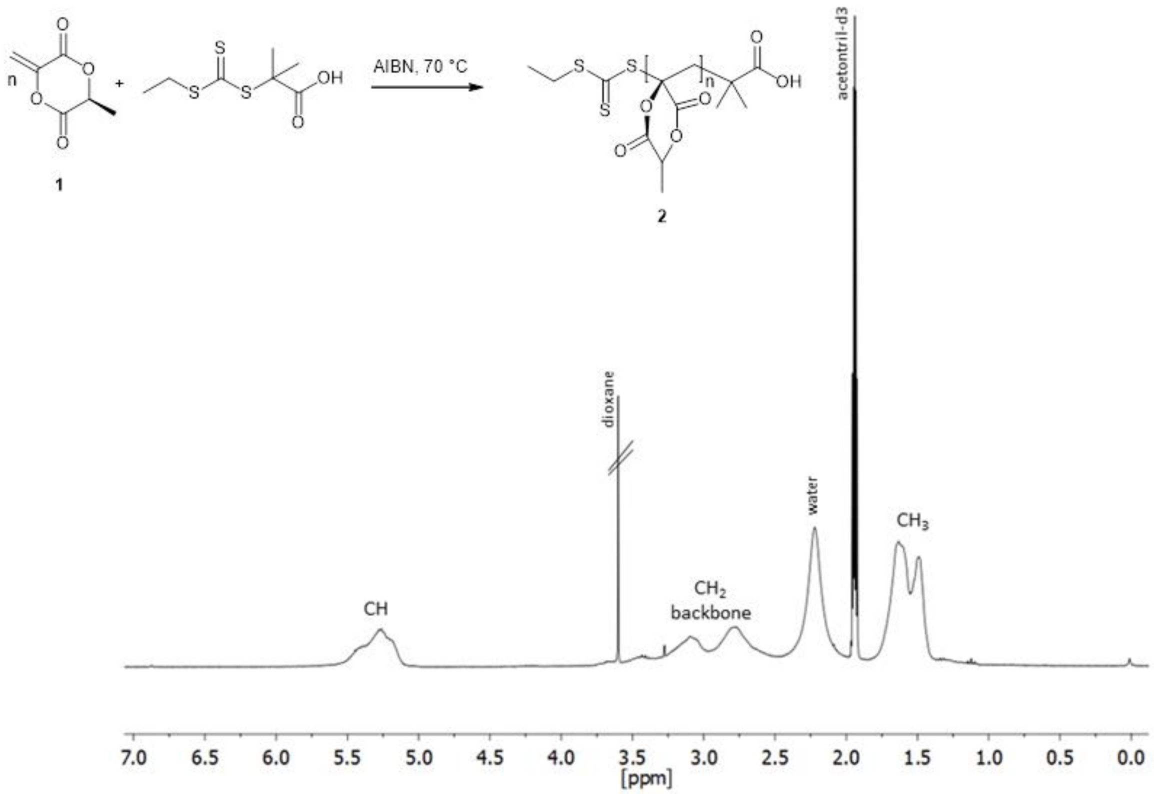

Figure S20: ^{1}H NMR of poly(MLA) of the RAFT run 4 (Table 4).

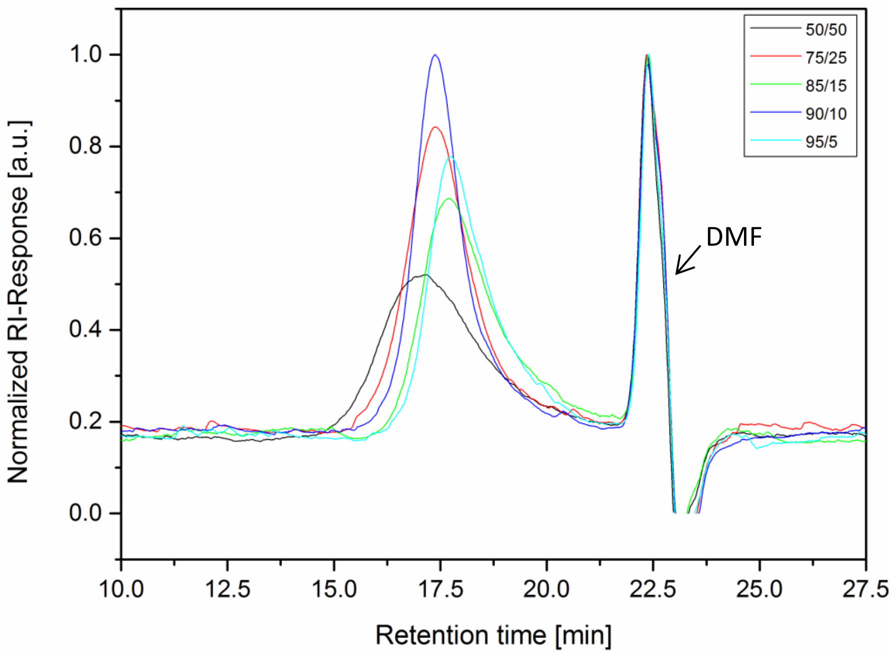

Figure S21: SEC traces for the copolymerization of DMA and MLA employing EMP with different DMA/MLA ratios (run 12 to run 17, monomer mixture/EMP/AIBN (100/0.5/0.0625), 80 wt % 1,4-dioxane, 70 °C).

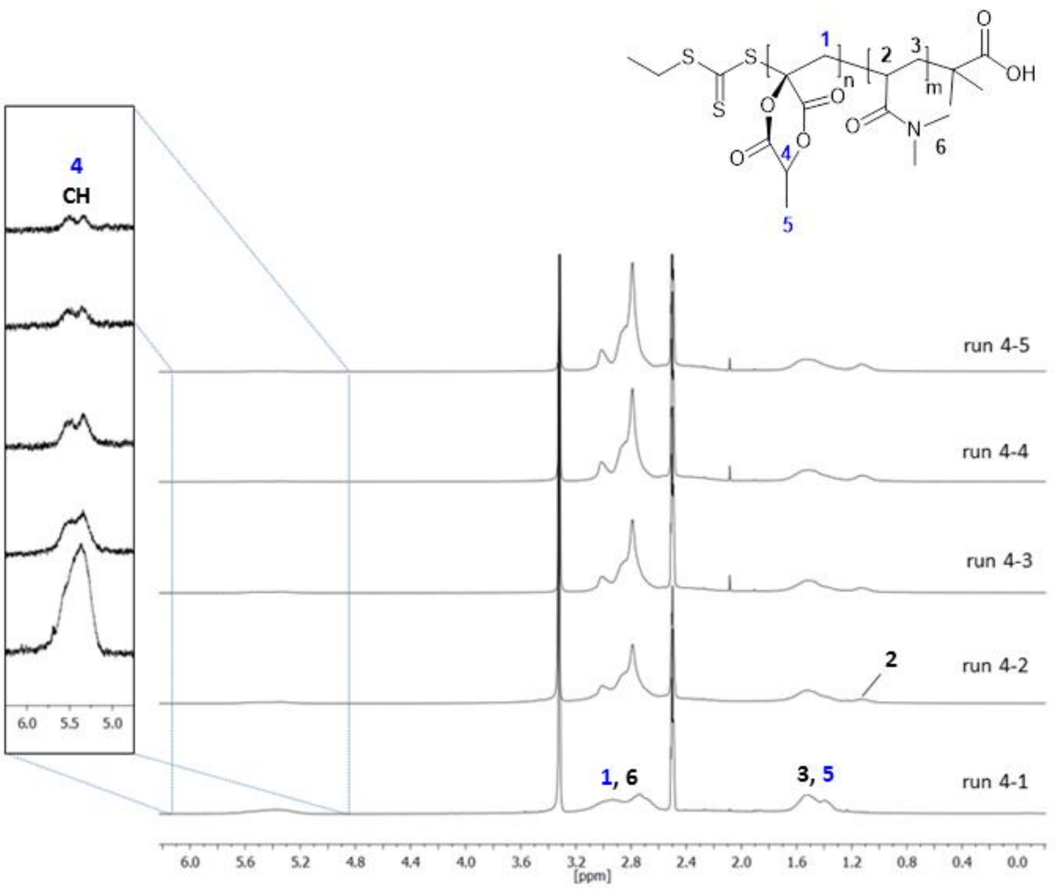

Figure S22: ^{1}H NMR spectra of poly(*N*,*N*-dimethylacrylamide-*co*-methylenelactide) of run 12 to run 17 (Table 4) with zoomed CH signals of the MLA unit.

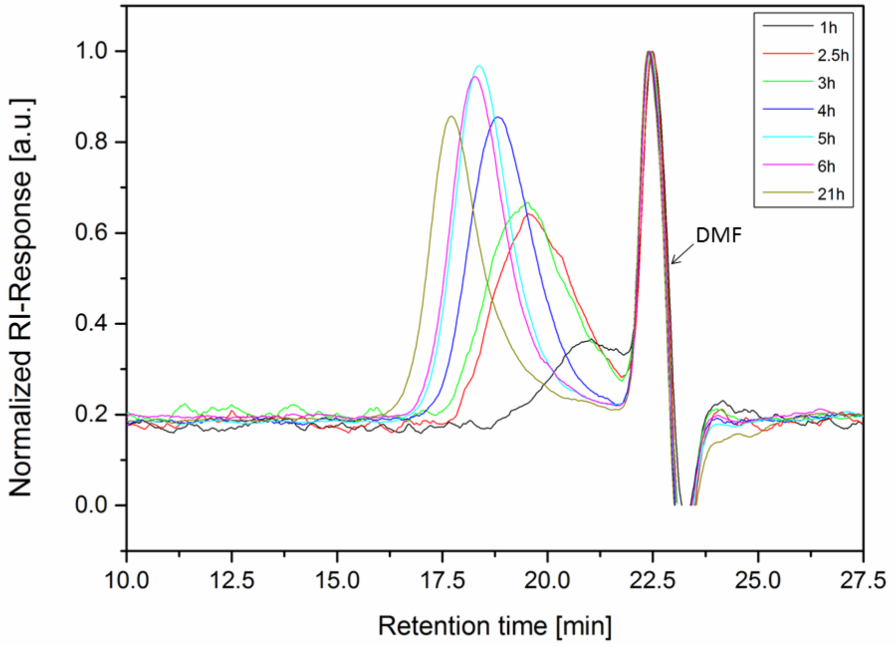

Figure S23: SEC traces from the kinetic of the copolymerization of DMA and MLA employing EMP with a ratio of 90/10 run 15 (monomer mixture/EMP/AIBN (100/0.5/0.0625), 80 wt % 1,4-dioxane, 70 °C).

Table S8: Results of the kinetic experiment of the RAFT copolymerization of DMA and MLA in a ratio of 90/10 run 15 using EMP (monomer mixture/ EMP/AIBN (100/0.5/0.0625), 80 wt % 1,4-dioxane, 70 °C).

time [h]	conv. [a] [%] MLA/DMA		$M_{n, theo.}$ [g mol^{-1}]	M_n [b] [g mol^{-1}]	$Đ$ [b]
**0.5**	3.8	(-7.3)	c)	c)	c)
**1**	24.2	2.1	1 288	4 040	1.20
**2.5**	70.3	15.2	4 942	7 289	1.35
**3**	78.8	15.0	5 143	8 024	1.35
**4**	100	33.7	9 078	11 618	1.28
**5**	100	43.9	10 908	16 214	1.23
**6**	100	50.5	12 072	17 271	1.23
**21**	100	93.8	19 806	23 498	1.23

a) Based on NMR-data.
b) Determined by PS-calibrated SEC.
c) Not enough polymer to precipitate.

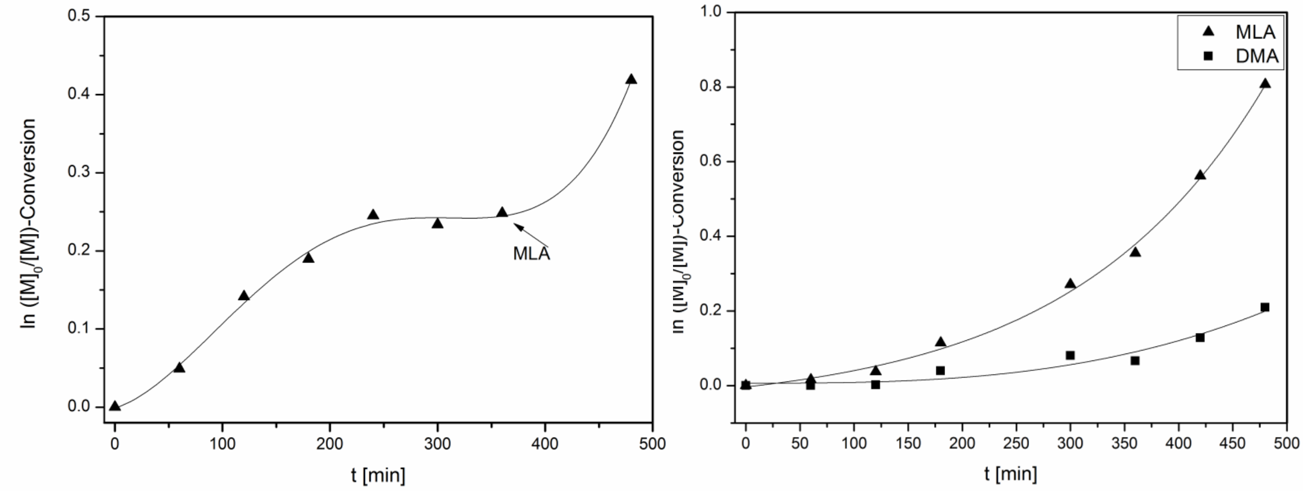

Figure S24: Both kinetic plot for the RAFT copolymerization of MLA and DMA for the ratio 75/25 (run 13, Table 5) employing EMP. On the left the first kinetic and on the right the repetition.

References

1. In order to avoid the formation of dimethylenelactide benzoyl peroxide was added dropwise via a syringe pump over 1.5 hours.
2. Jing, F.; Hillmyer, M. A. *J. Am. Chem. Soc.* **2008,** *130,* 13826-13827.
3. Loubat, C.; Batt-Coutrot, D.; Guyot, B.; Robin, J. J.; Boutevin, B. *Polym. Int* **2005,** *54,* 1557-1563.

4. Stöhr, O.; Ritter, H. *Macromol. Chem. Phys.* **2014,** *215,* 426-430.
5. Schmidt, B. V. K. J.; Hetzer, M.; Ritter, H.; Barner-Kowollik, C. *Macromolecules* **2011,** *44,* 7220-7232.
6. Convertine, A. J.; Benoit, D. S. W.; Duvall, C. L.; Hoffman, A. S.; Stayton, P. S. *J. Controlled Release* **2009,** *133,* 221-229.
